# Supplementary material for: LAG3 is not expressed in human and murine neurons and does not modulate α‐synucleinopathies
Source: EMBO Mol Med. 2021 Jul 26;13(9):e14745. doi: 10.15252/emmm.202114745 (PMC8422075; doi:10.15252/emmm.202114745)
Supplement: Supplementary file 1 — Expanded View Figures PDF [file EMMM-13-e14745-s006.pdf]

Expanded View Figures

Figure EV1. LAG3 expression in human and murine cells.

A Expression levels of cellular markers and control genes in human NSC-derived neuronal cultures assessed by scRNAseq. Data shown from 5,476 unique analysed cells from one out of two independent biological replicates.

B Immunofluorescence images of iPSC-derived dopaminergic neuronal cultures from controls and PD patients with N370S mutations in GBA. Staining for DAPI and tyrosine hydroxylase (TH). Scale bar represent 100 μm.

C Western blotting of brain homogenates from LAG3 KO and WT mice, with activated murine T cells as controls. Anti-LAG3 antibody LSB15026 was used.

Source data are available online for this figure.

© 2021 The Authors

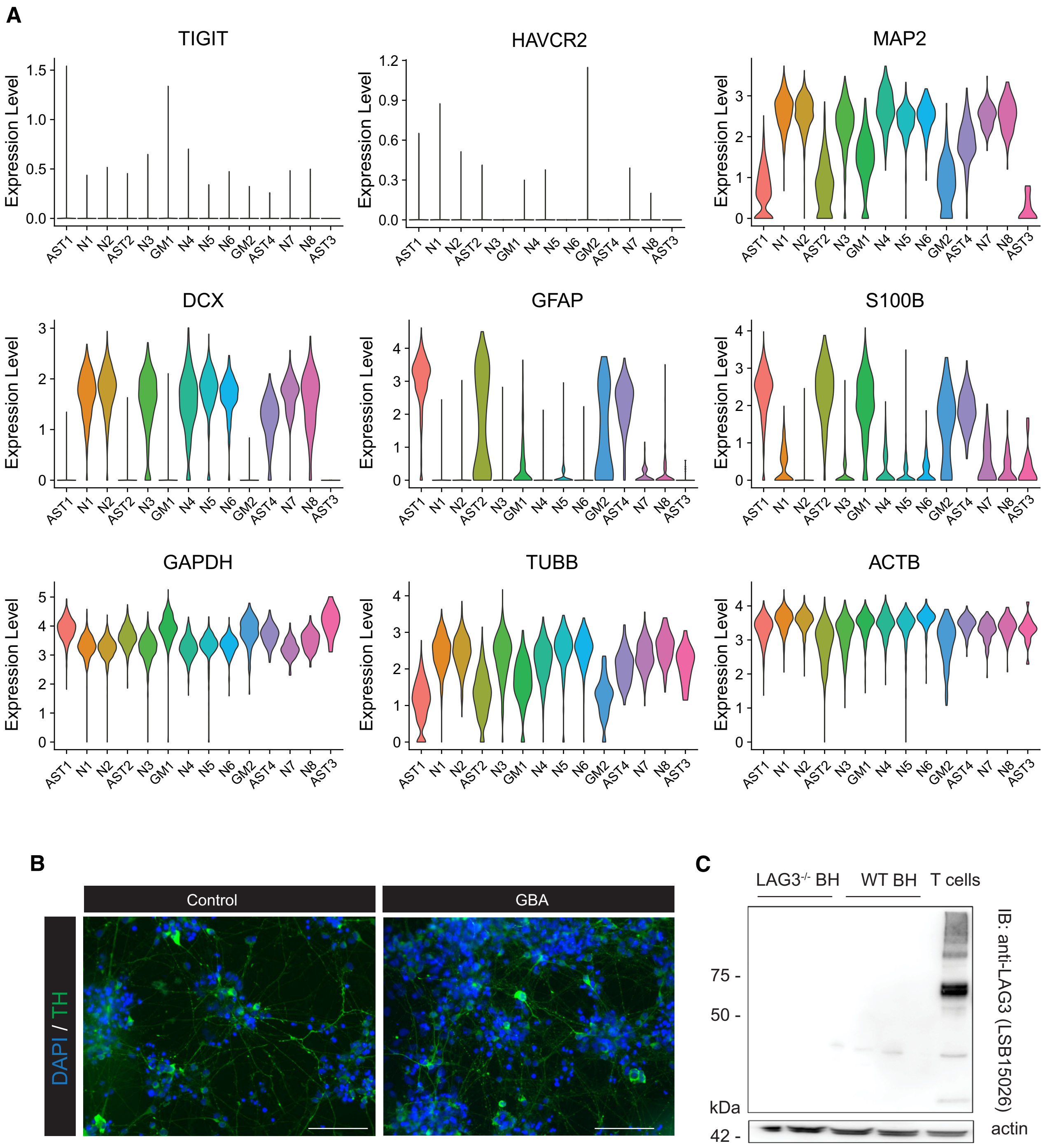

Figure EV1.

© 2021 The Authors

Figure EV2. IP enrichment and scRNAseq analysis of Lag3 transcript levels in murine samples.

A, B IP enrichment of murine brain homogenates (A) and cultures (B). The enrichment was performed with anti-LAG3 antibody (4-10-C9), and the immunoblot was done with LSB15026.

C Uniform Manifold Approximation and Projection (UMAP) visualization of cell clusters identified by scRNAseq of 76,305 cells derived from the mouse midbrain and striatum, with major cell types labelled.

D Feature plot showing distribution of Lag3 transcripts in the identified mouse cell types from the midbrain and striatum area.

E Lag3 transcript levels after striatal injections of PBS or LPS. LPS treatment decreases detectable transcripts, while the basal expression is already extremely low.

Source data are available online for this figure.

© 2021 The Authors

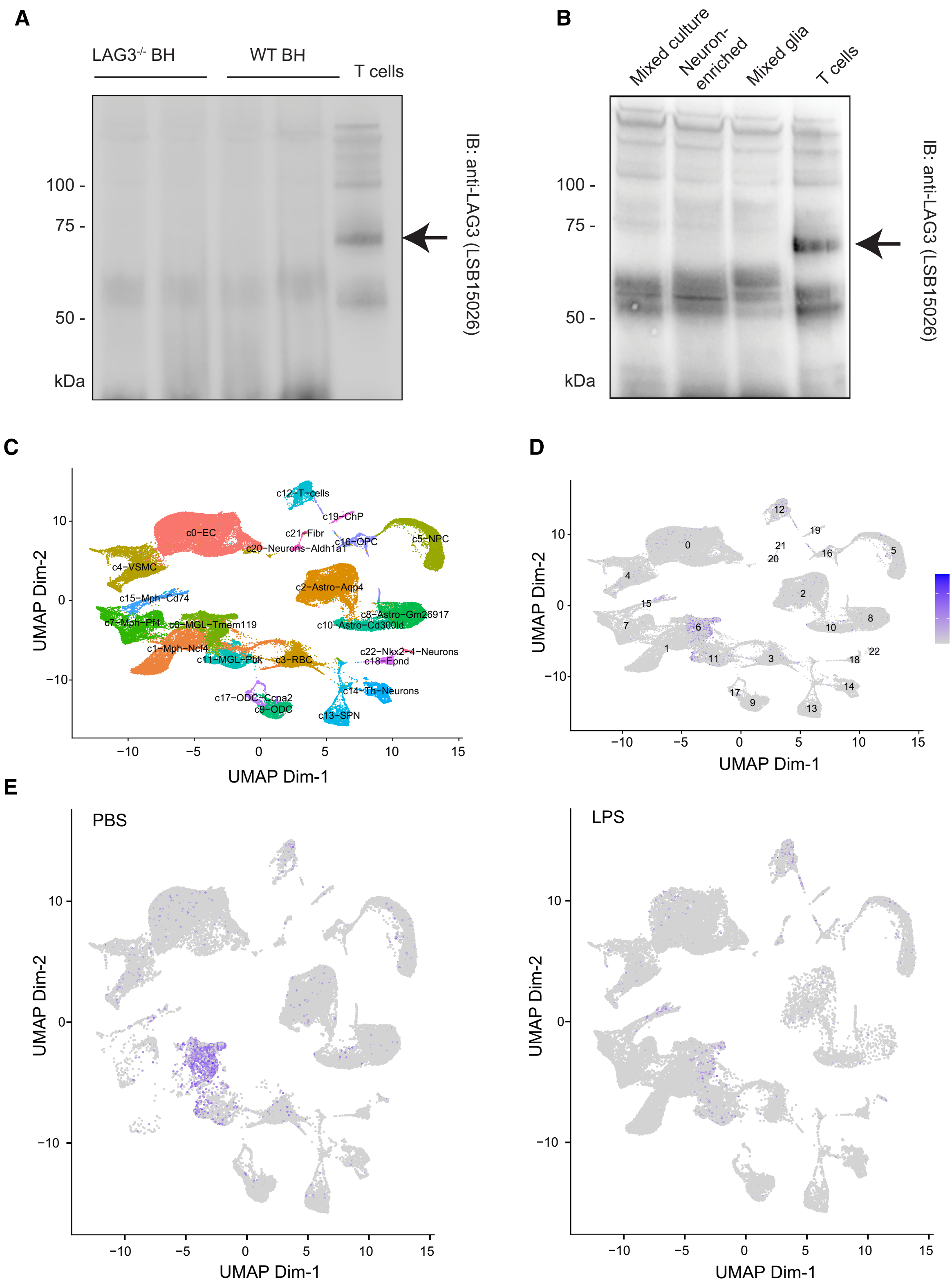

Figure EV2.

© 2021 The Authors

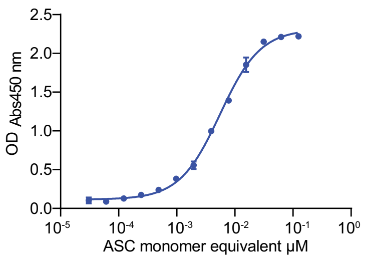

Figure EV3. Binding of anti-ASC antibody to ASC filaments in indirect ELISA.

Serially diluted ASC filaments, directly bound to the plate, were recognized using an anti-ASC antibody in indirect ELISA. Shown are means ± SD of technical replicates.

Source data are available online for this figure.

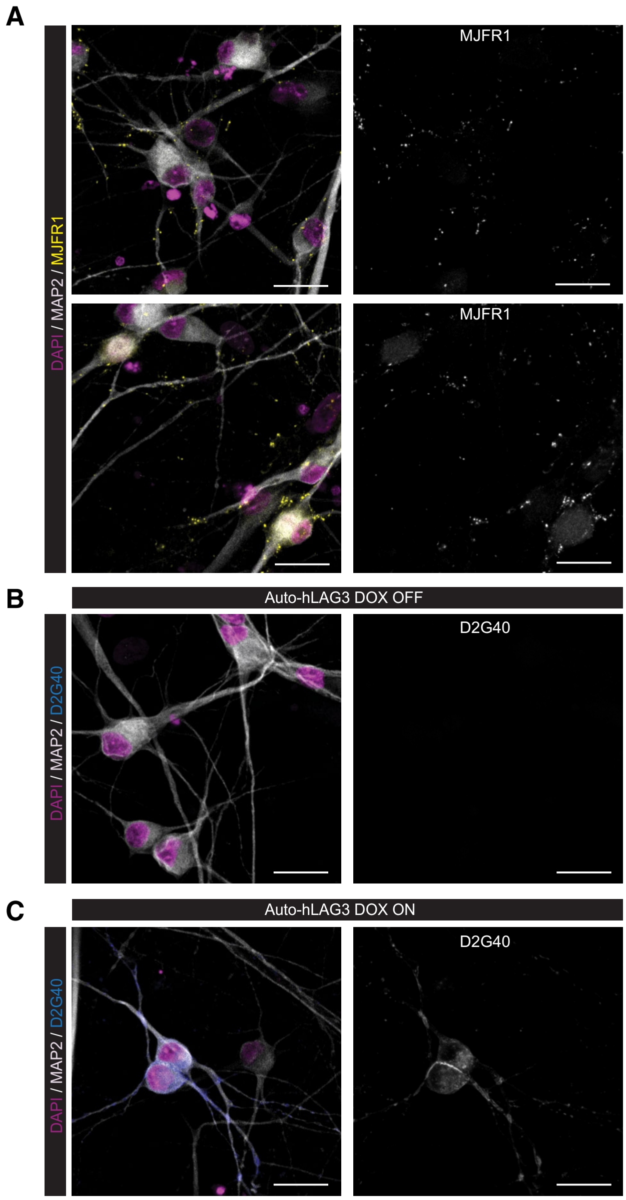

Figure EV4. Expression of α-synuclein and LAG3 in NSC-derived neural cultures.

- A Neurons ($MAP2^{+}$ cells) in human neural cultures express α-synuclein (MJFR1).
- B Human neural cultures transduced with Auto-hLAG3 LVs do not show any positivity for LAG3 (17B4) in DOX OFF condition.
- C Upon inducing the expression of hLAG3 (DOX ON condition), transgenic neurons display LAG3 positivity (17B4), while non-transduced cells remain negative. Data information: Scale bars 25 μm.

Source data are available online for this figure.

© 2021 The Authors

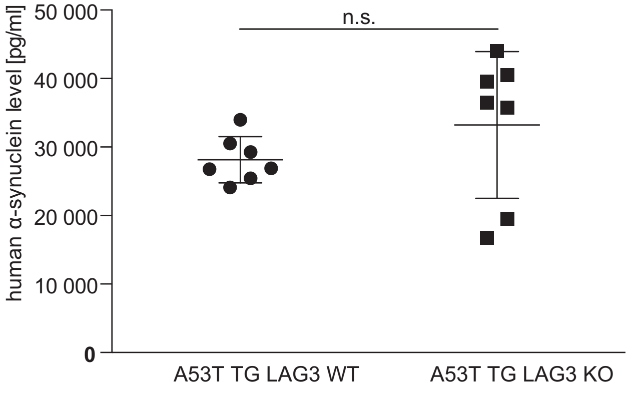

Figure EV5. Quantification of α-synuclein in murine CSF.

Levels of α-synuclein in murine CSF quantified using SIMOA. No significantly altered expression levels of human α-synuclein could be identified (Mann–Whitney test). Shown are all dots as well as their means and the standard deviation. Average age of A53T α-synuclein TG LAG3$^{+/+}$ (LAG3 WT; $n = 7$) was 9.1 months; 8.0 months for A53T α-synuclein TG LAG3$^{-/-}$ (LAG3 KO; $n = 7$).

Source data are available online for this figure.

© 2021 The Authors
