# Supplementary figures and images for: LAG3 is not expressed in human and murine neurons and does not modulate α‐synucleinopathies
Source: EMBO Mol Med. 2021 Jul 26;13(9):e14745. doi: 10.15252/emmm.202114745 (PMC8422075; doi:10.15252/emmm.202114745)

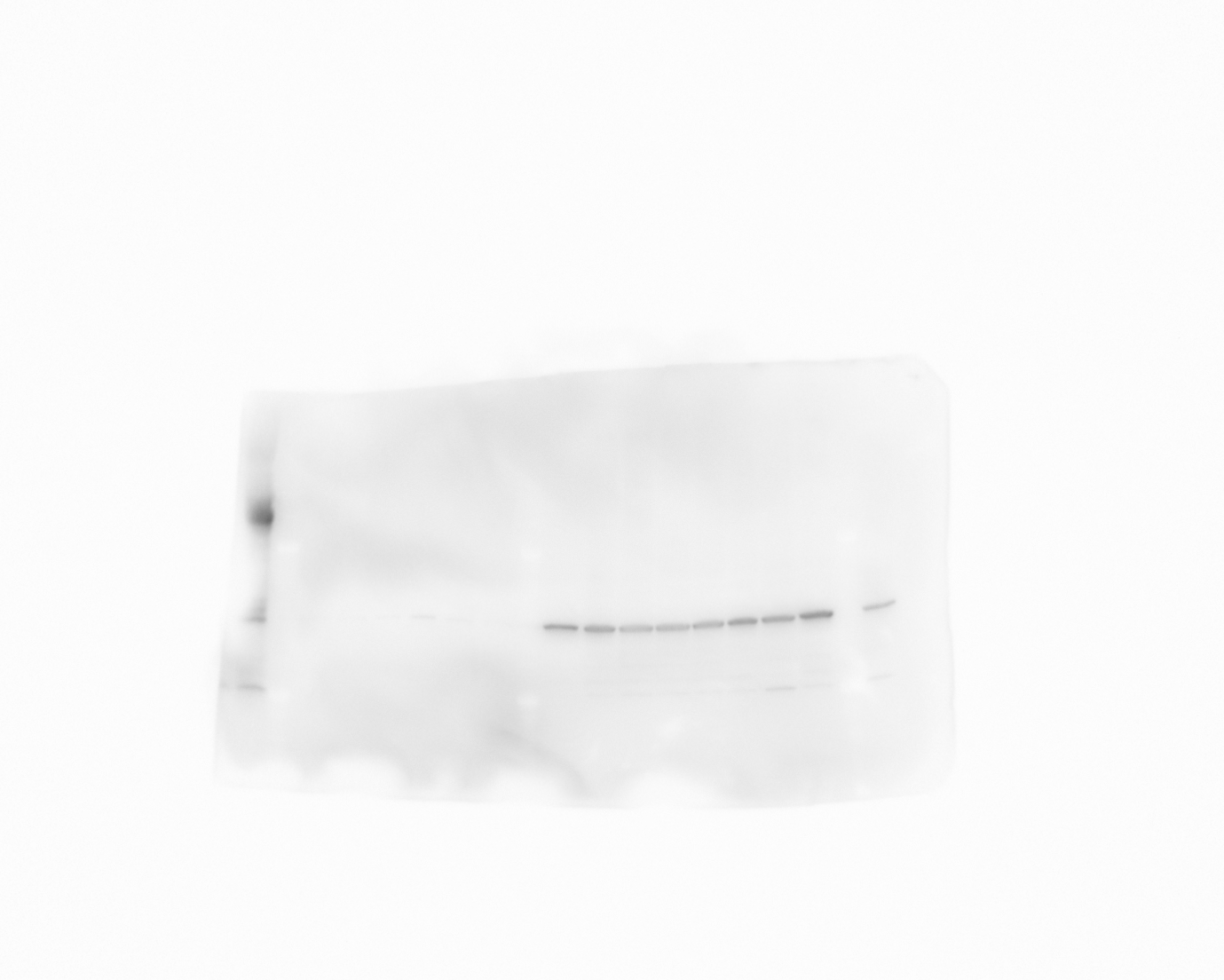

Supplement: Supplementary file 4 — Source Data for Figure 1 [file EMMM-13-e14745-s001.zip › Fig1/1F/BACT.tif]

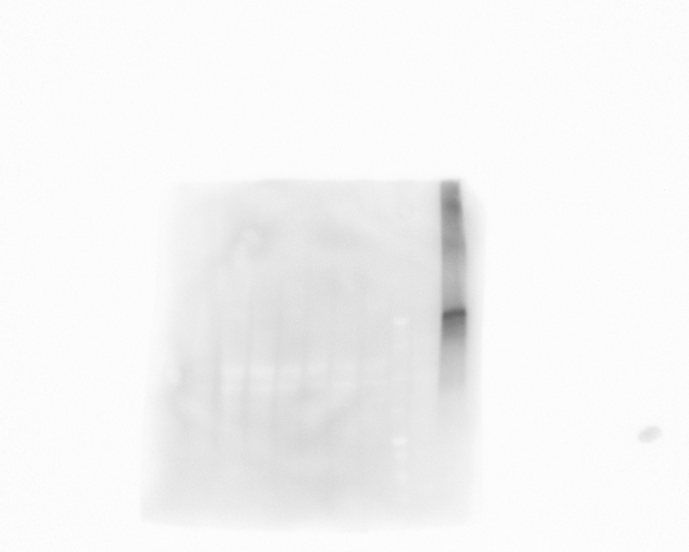

Supplement: Supplementary file 4 — Source Data for Figure 1 [file EMMM-13-e14745-s001.zip › Fig1/1F/LAG3.tif]

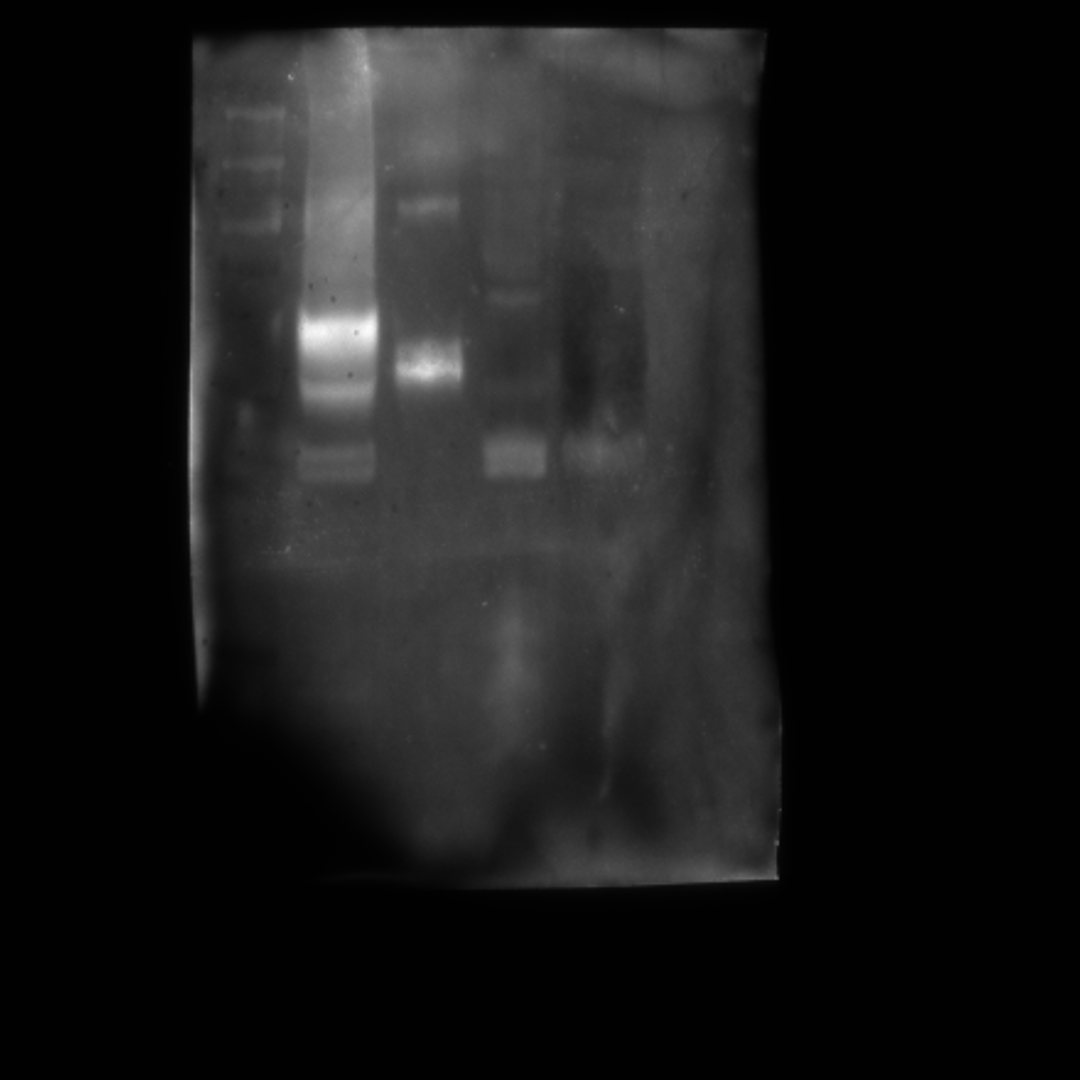

Supplement: Supplementary file 4 — Source Data for Figure 1 [file EMMM-13-e14745-s001.zip › Fig1/1B/Mixed mLag3-recmLAG3-KO-Mixed huLag3-rec HuLag3_actin_3s.Tif]

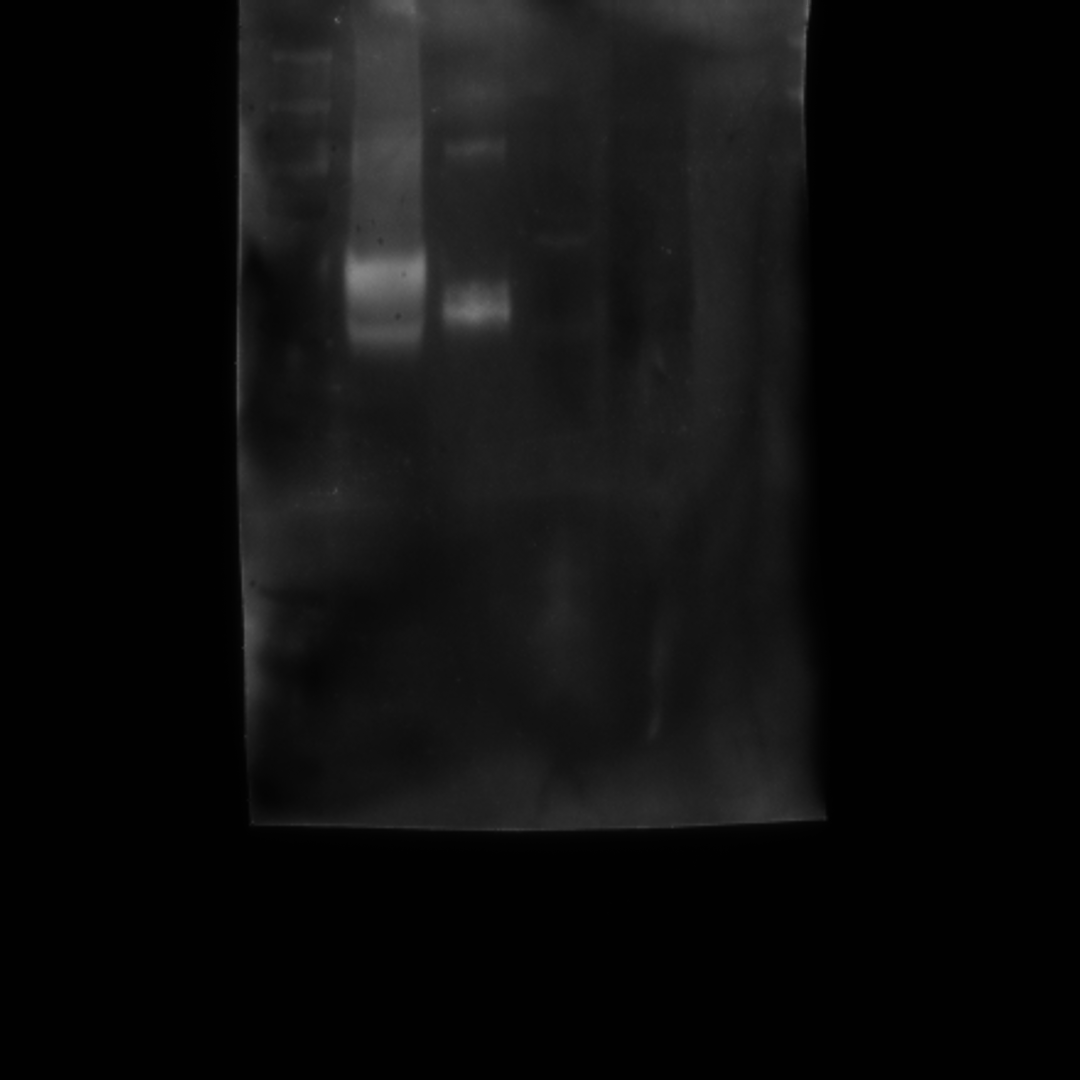

Supplement: Supplementary file 4 — Source Data for Figure 1 [file EMMM-13-e14745-s001.zip › Fig1/1B/Mixed mLag3-recmLAG3-KO-Mixed huLag3-rec HuLag3_410C9_2s.Tif]

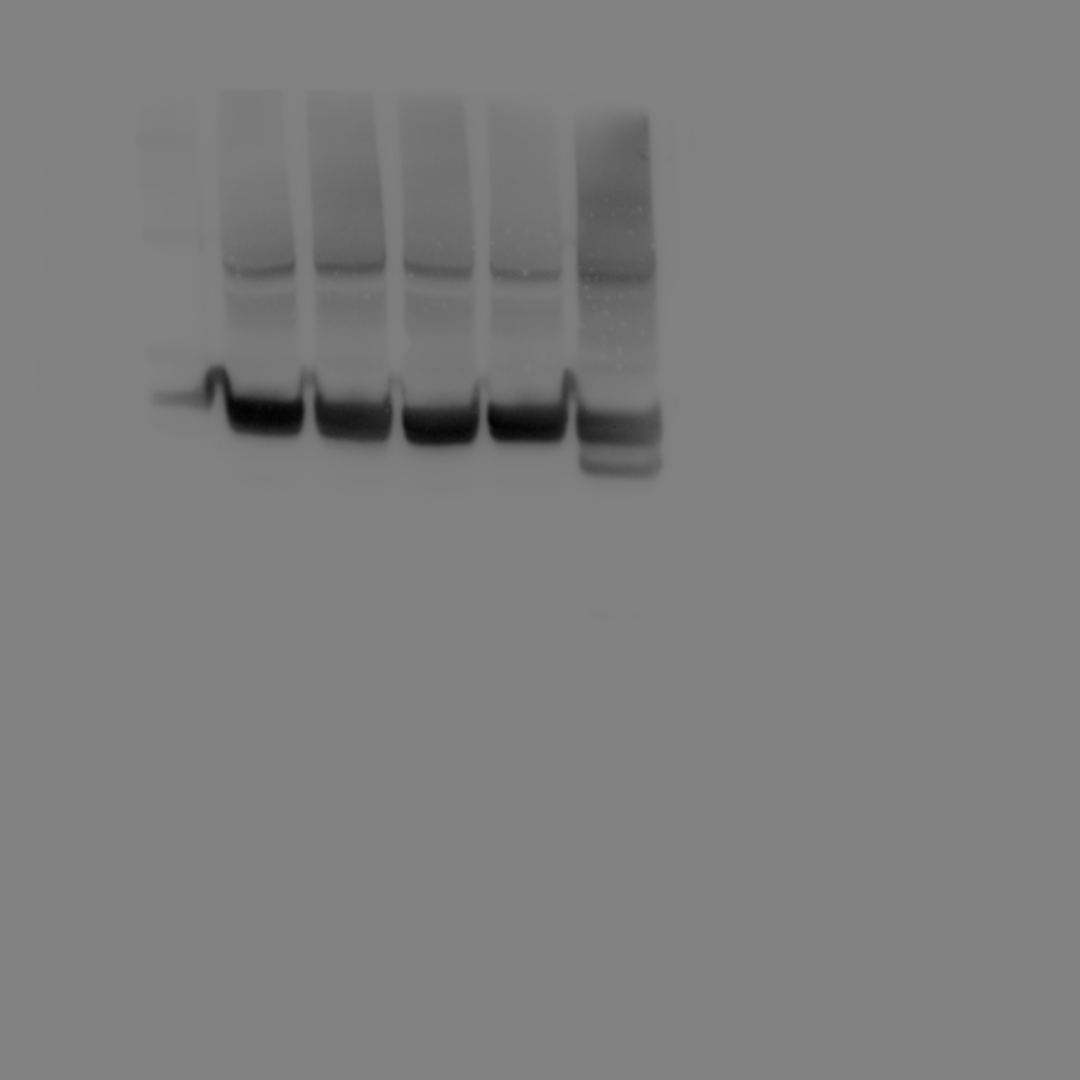

Supplement: Supplementary file 4 — Source Data for Figure 1 [file EMMM-13-e14745-s001.zip › Fig1/1D/Transduction control-transduced no dox - transduced dox - wt - t cells_actin.Tif]

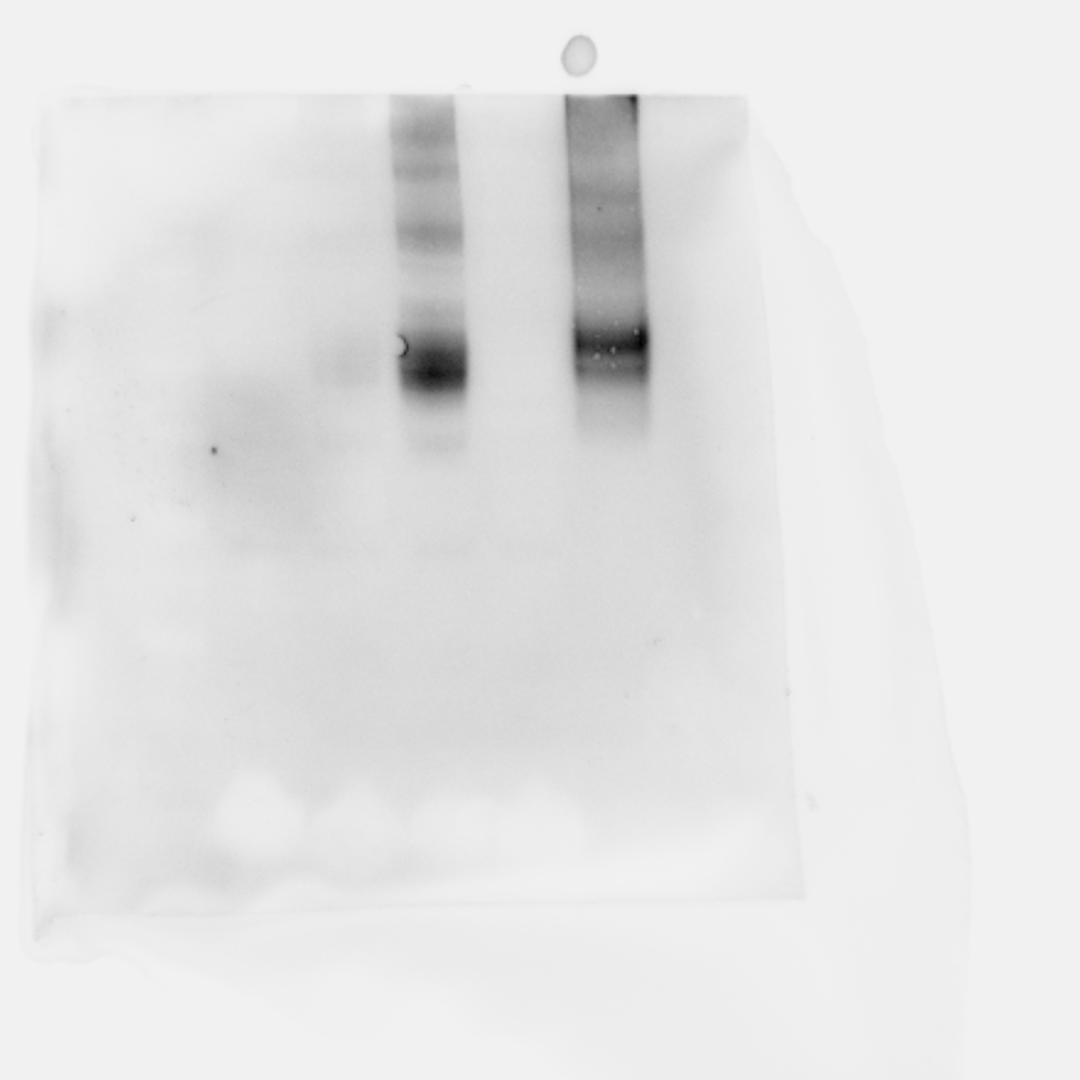

Supplement: Supplementary file 4 — Source Data for Figure 1 [file EMMM-13-e14745-s001.zip › Fig1/1D/Transduction control-transduced no dox - transduced dox - wt - t cells_D2g40_1min8s.Tif]

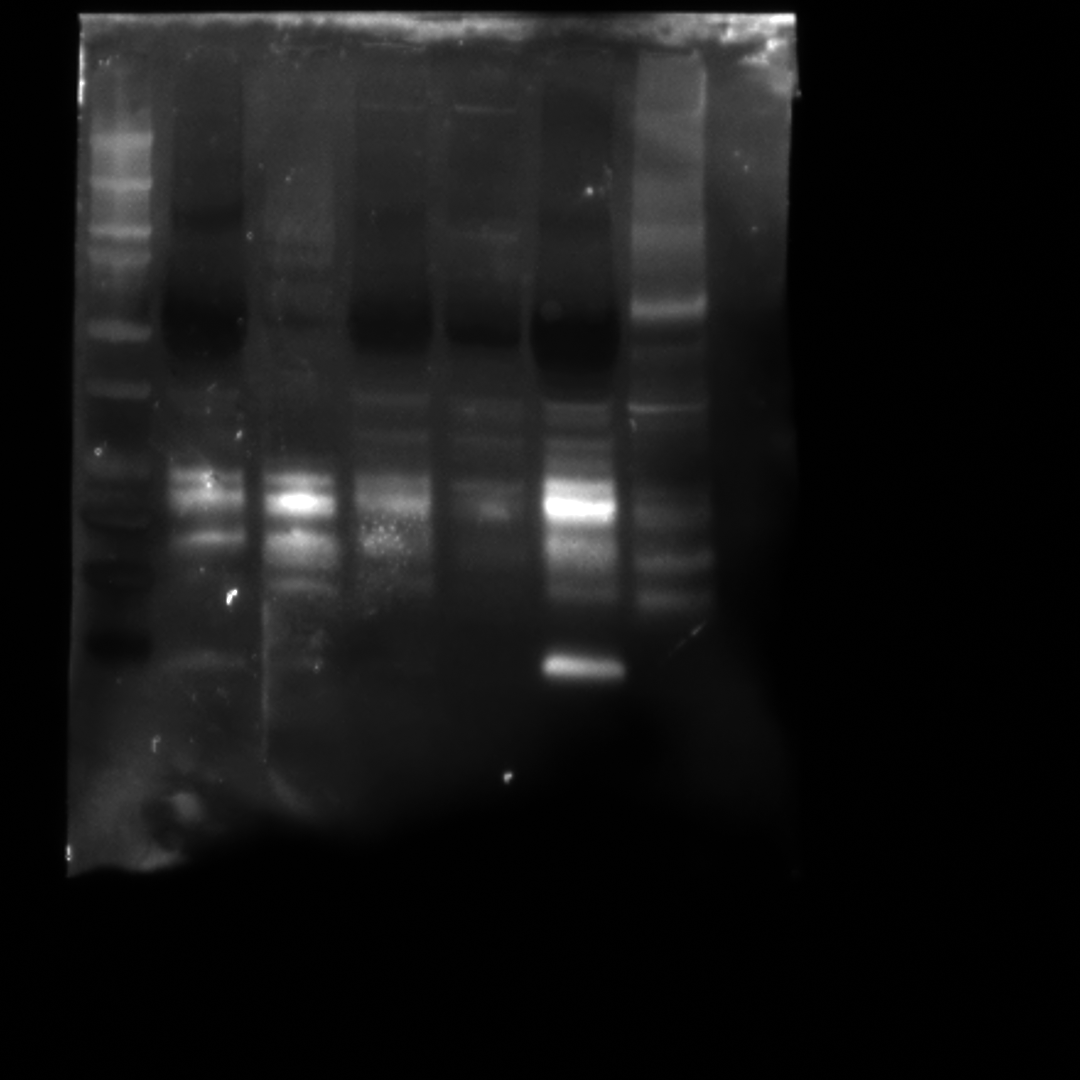

Supplement: Supplementary file 4 — Source Data for Figure 1 [file EMMM-13-e14745-s001.zip › Fig1/1C/shsy5y-hek-u251-chp-hmc-tcells_d2g40_3s.Tif]

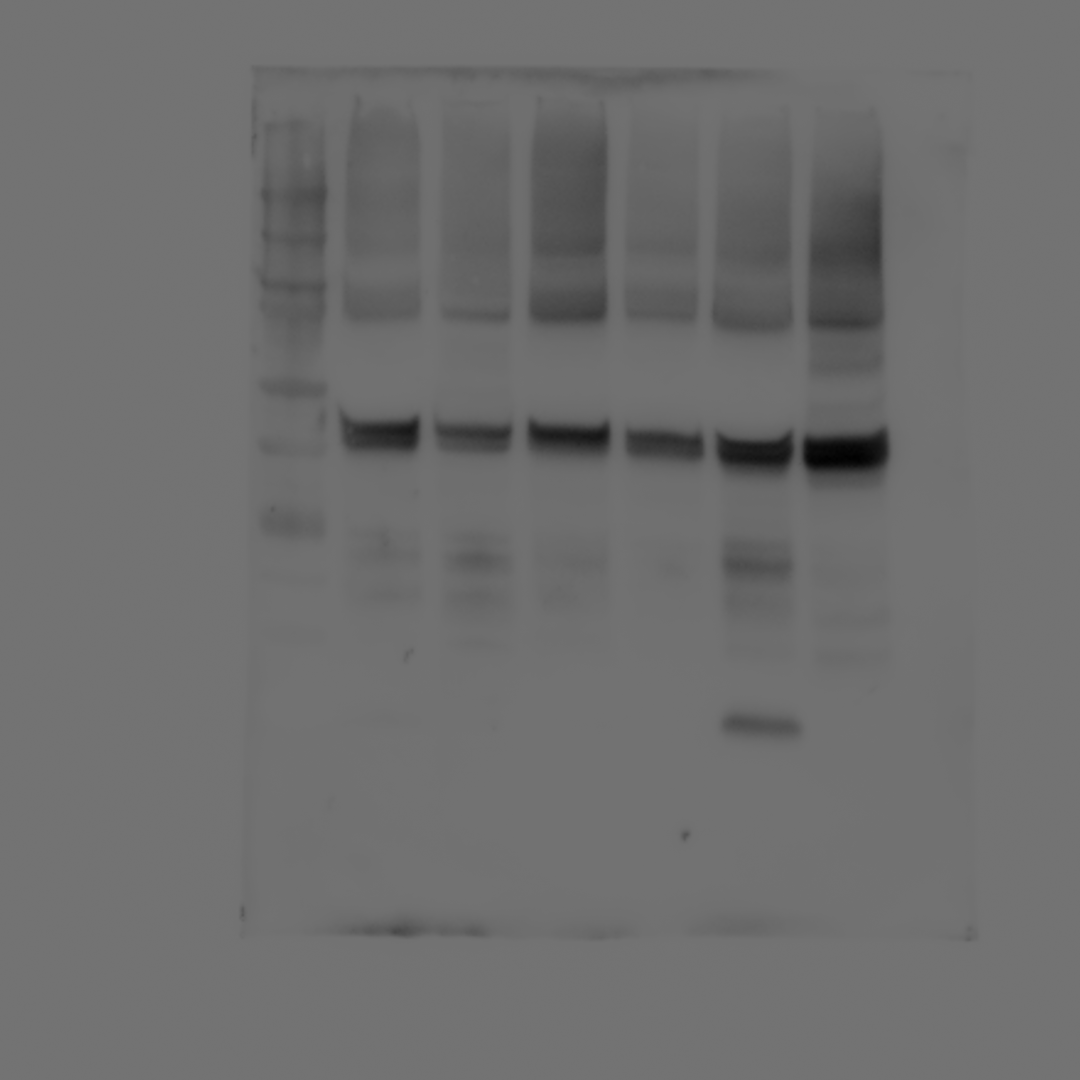

Supplement: Supplementary file 4 — Source Data for Figure 1 [file EMMM-13-e14745-s001.zip › Fig1/1C/shsy5y-hek-u251-chp-hmc-tcells_actin_3s.Tif]

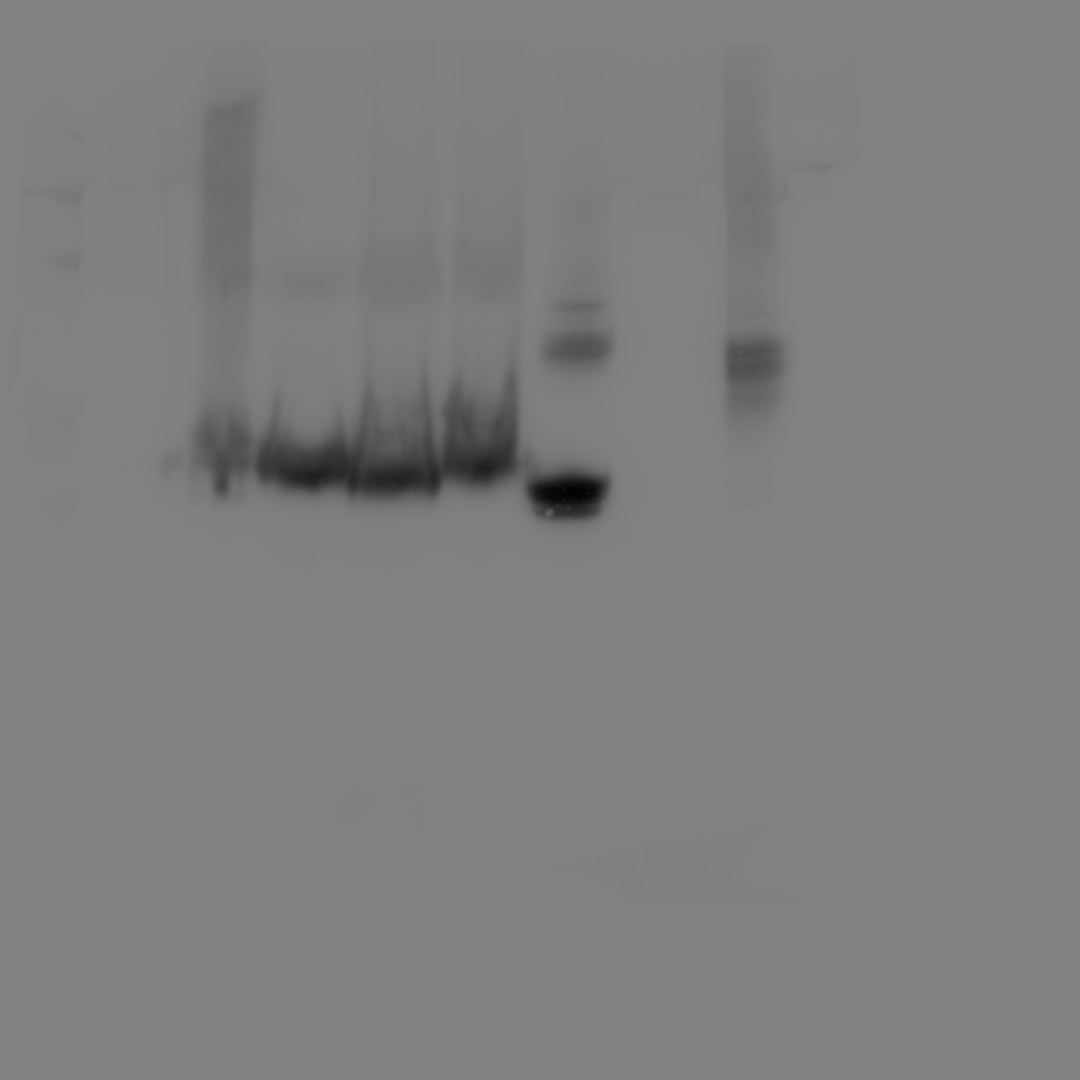

Supplement: Supplementary file 5 — Source Data for Figure 2 [file EMMM-13-e14745-s004.zip › Fig2/2A/20-21-22-23-Tcells-empty_hekoe_actin.Tif]

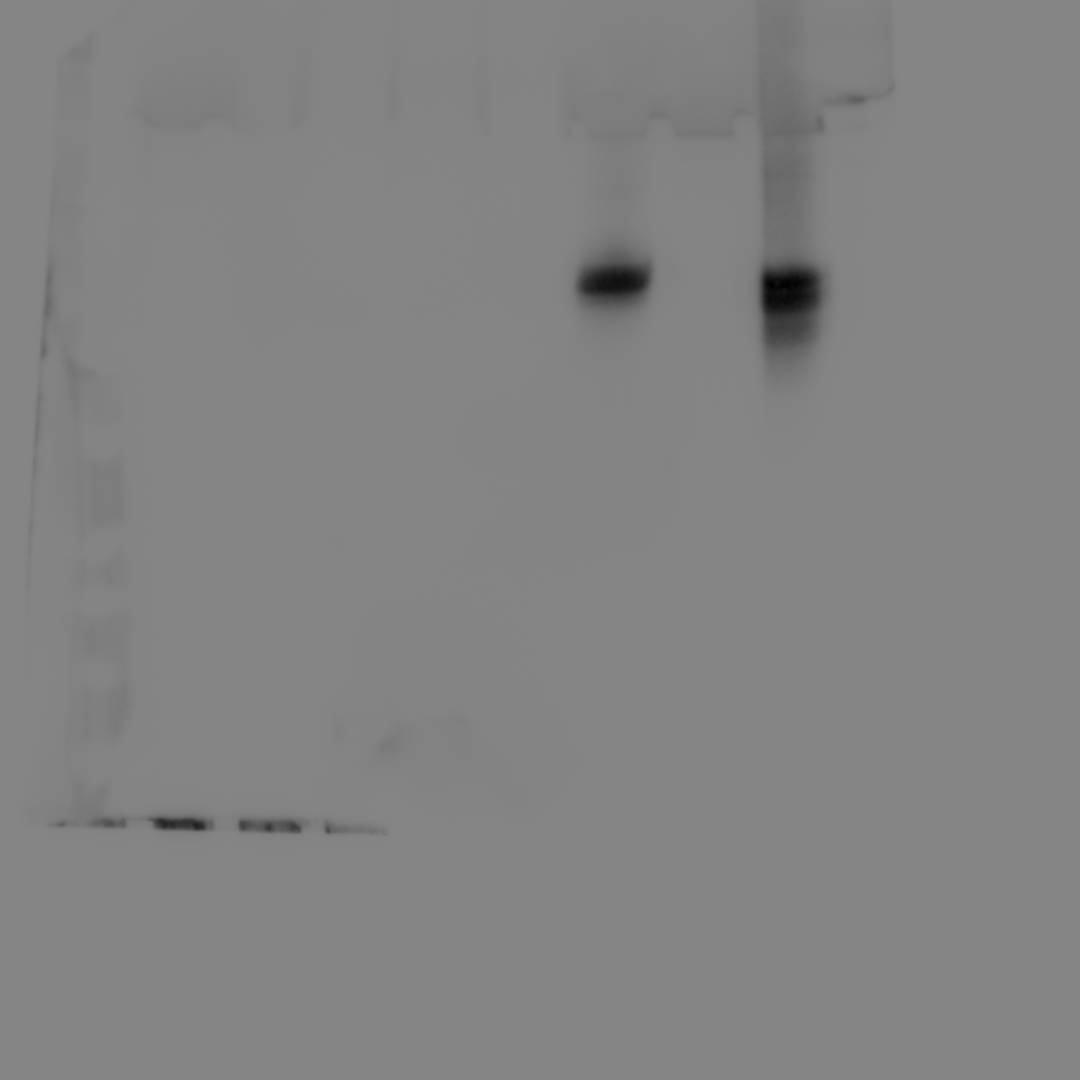

Supplement: Supplementary file 5 — Source Data for Figure 2 [file EMMM-13-e14745-s004.zip › Fig2/2A/20-21-22-23-Tcells-empty_hekoe_4-10-C9_2s.Tif]

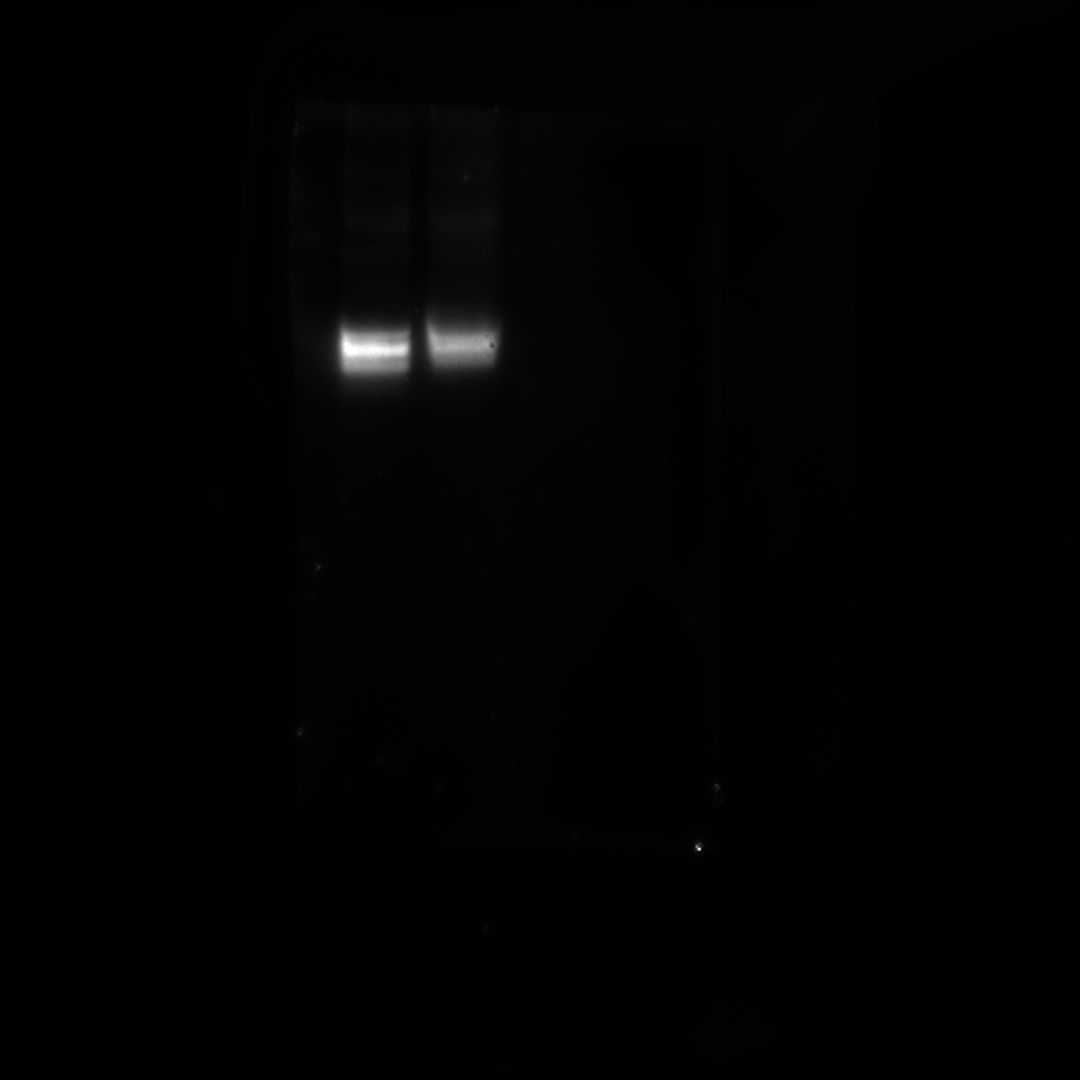

Supplement: Supplementary file 5 — Source Data for Figure 2 [file EMMM-13-e14745-s004.zip › Fig2/2B/Mixed-Neurons-glia-t cells_NeuN_17s.Tif]

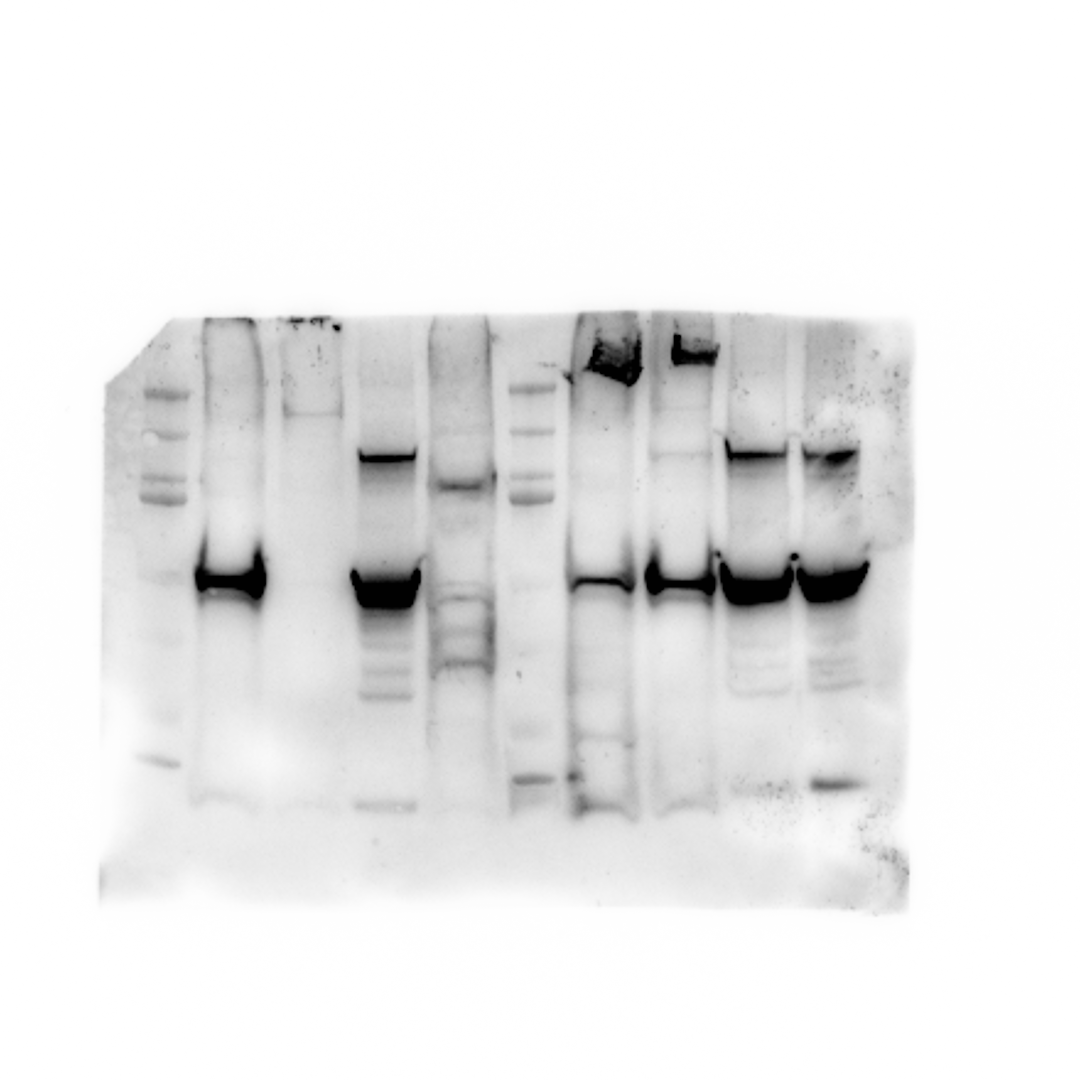

Supplement: Supplementary file 5 — Source Data for Figure 2 [file EMMM-13-e14745-s004.zip › Fig2/2B/Iba1 30s.Tif]

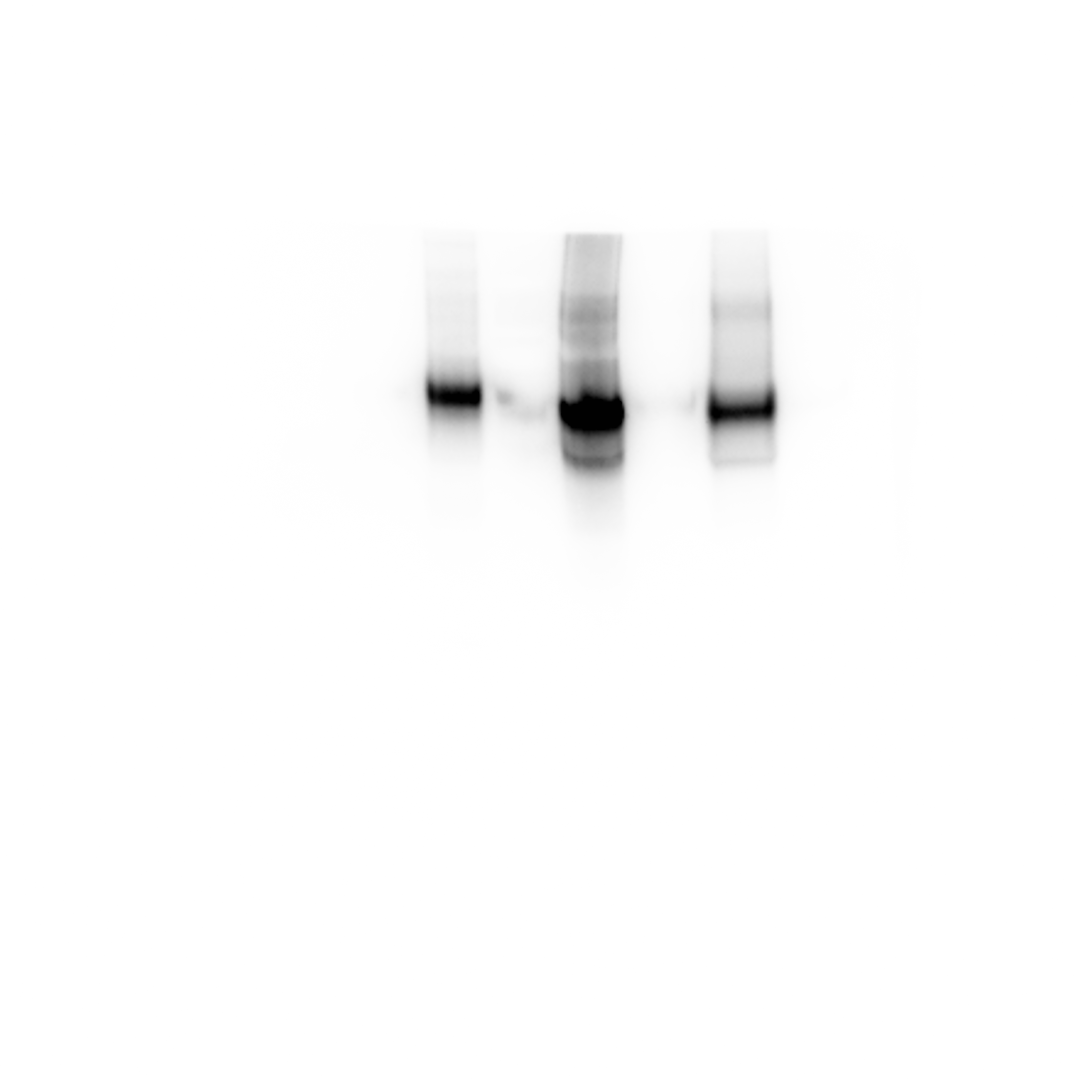

Supplement: Supplementary file 5 — Source Data for Figure 2 [file EMMM-13-e14745-s004.zip › Fig2/2B/lag3 2 1s.Tif]

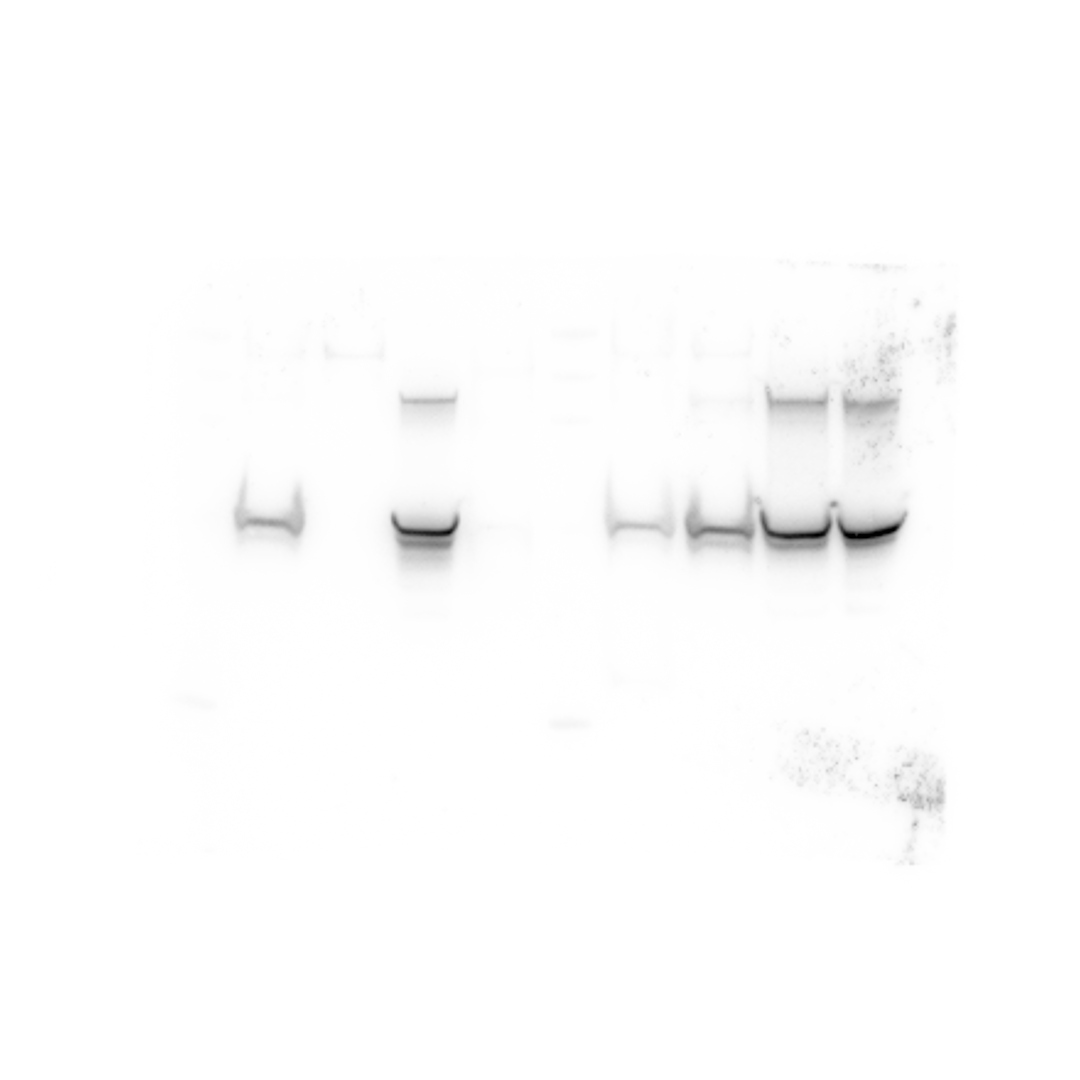

Supplement: Supplementary file 5 — Source Data for Figure 2 [file EMMM-13-e14745-s004.zip › Fig2/2B/GFAP 6s.Tif]

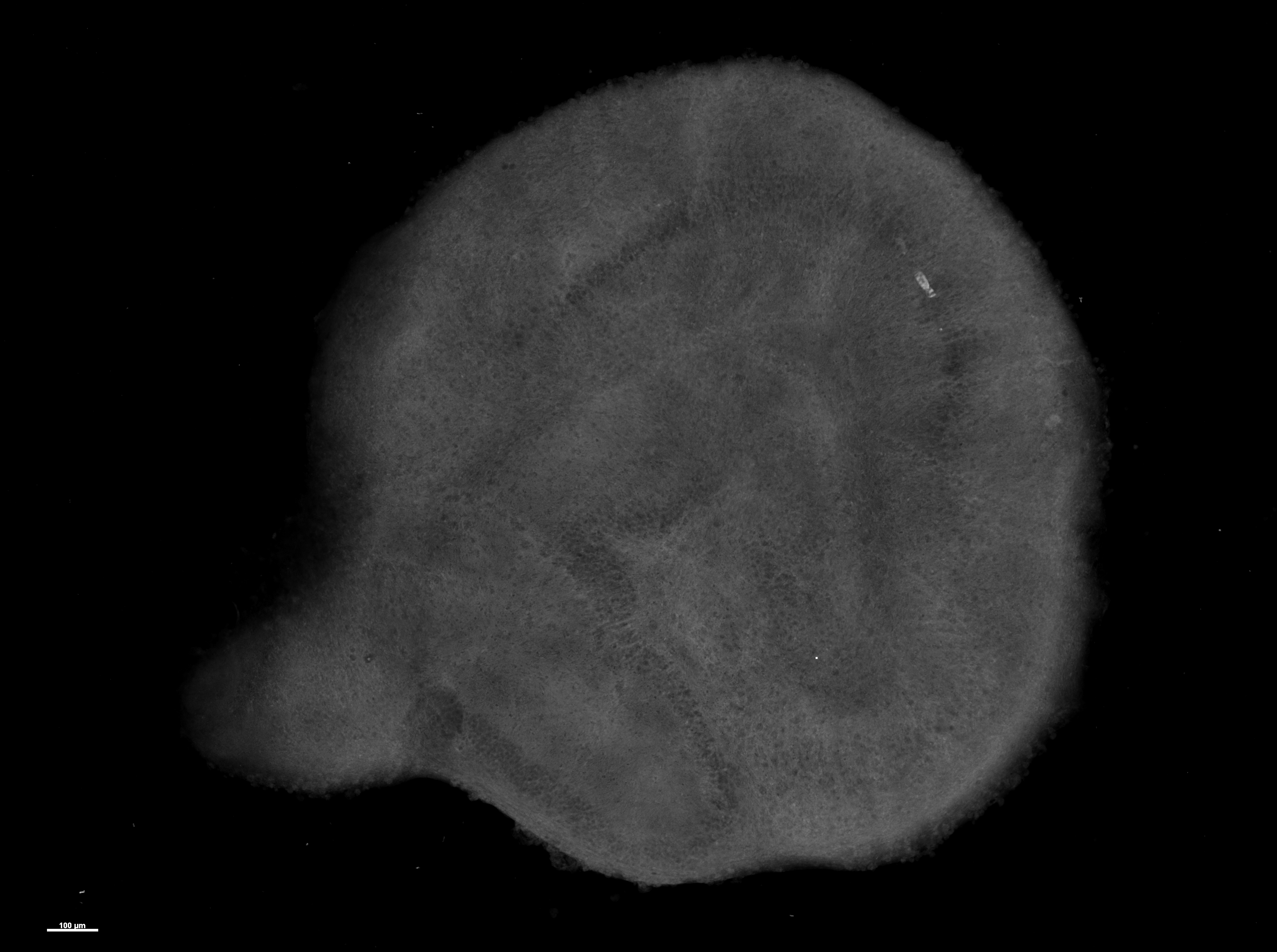

Supplement: Supplementary file 8 — Source Data for Figure 5 [file EMMM-13-e14745-s008.zip › Fig5/5C/20171206_lag3-exp_5-R2S2_ThioS+pS129-488_10x_(c1).TIF (green).tif]

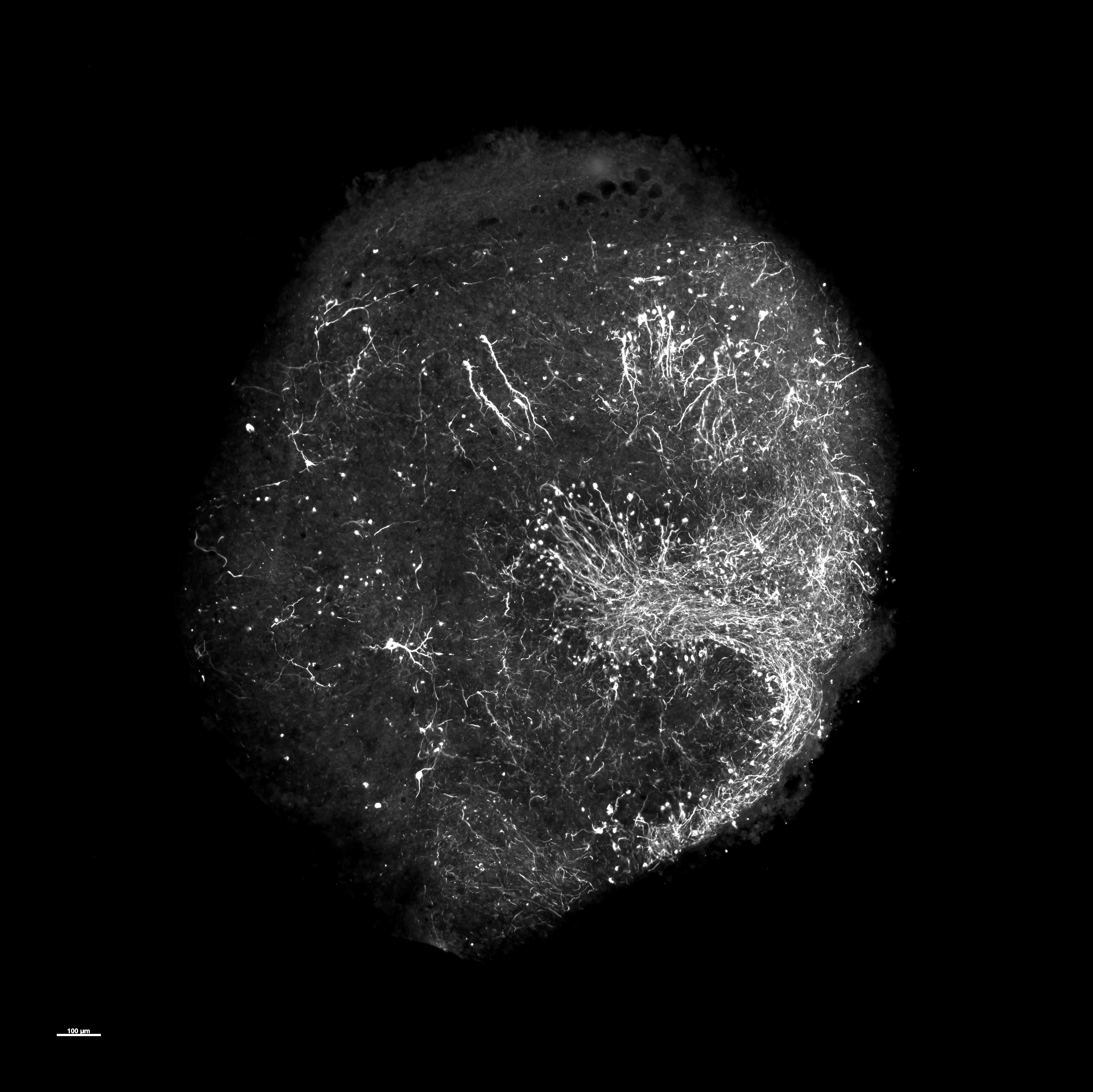

Supplement: Supplementary file 8 — Source Data for Figure 5 [file EMMM-13-e14745-s008.zip › Fig5/5C/20180426_LAG3-BLIND-8-r4s2_ThioS-FITC-700ms_pS129-Cy3-500ms_DAPI-30ms_10x_(DAPI+FITC+Cy5 5).TIF (red).tif]

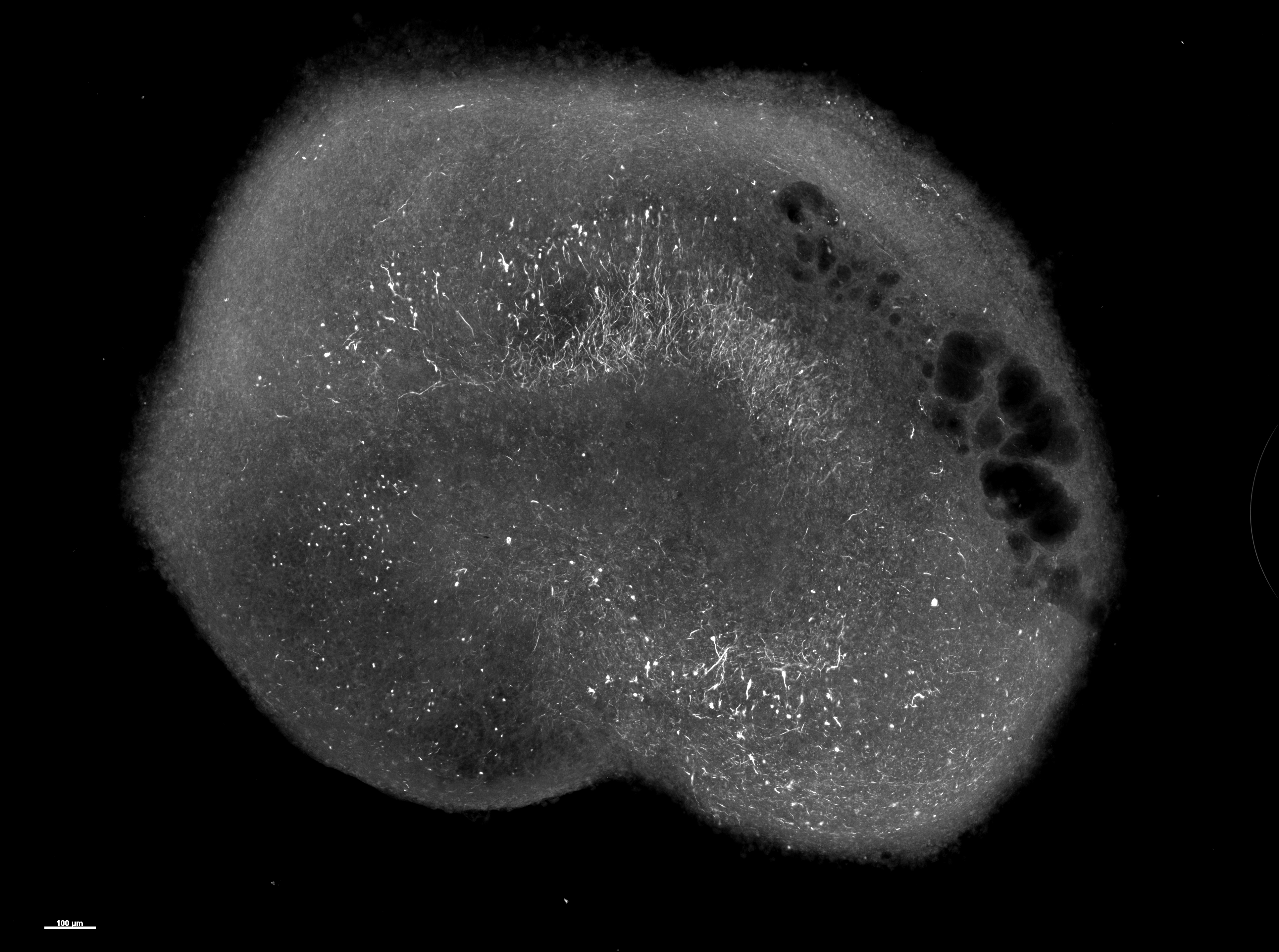

Supplement: Supplementary file 8 — Source Data for Figure 5 [file EMMM-13-e14745-s008.zip › Fig5/5C/20171205_lag3-exp_2-R5S3_ThioS+pS129-488_10x.TIF (green).tif]

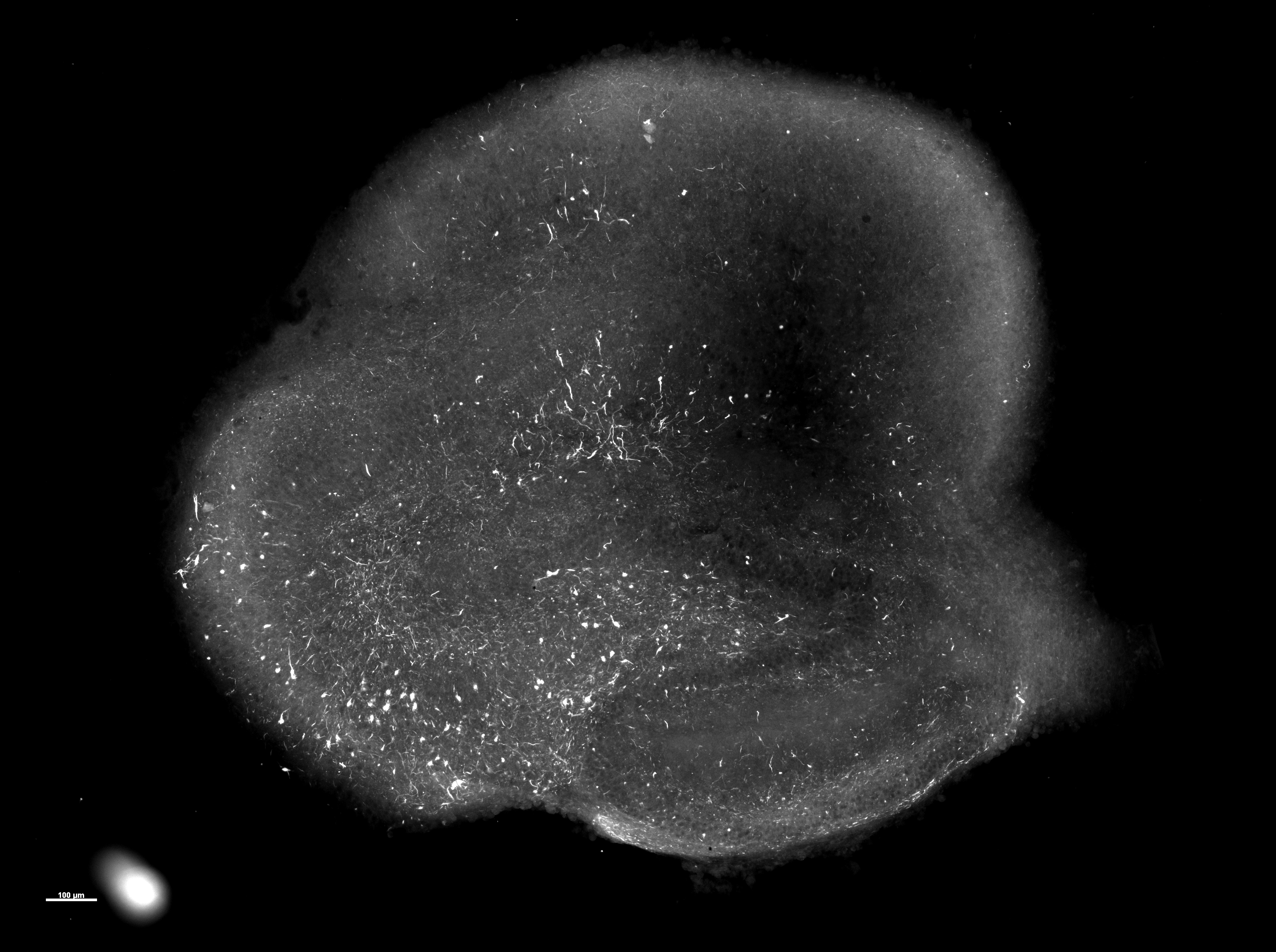

Supplement: Supplementary file 8 — Source Data for Figure 5 [file EMMM-13-e14745-s008.zip › Fig5/5C/20171205_lag3-exp_9-R1S3_ThioS+pS129-488_10x.TIF (green).tif]

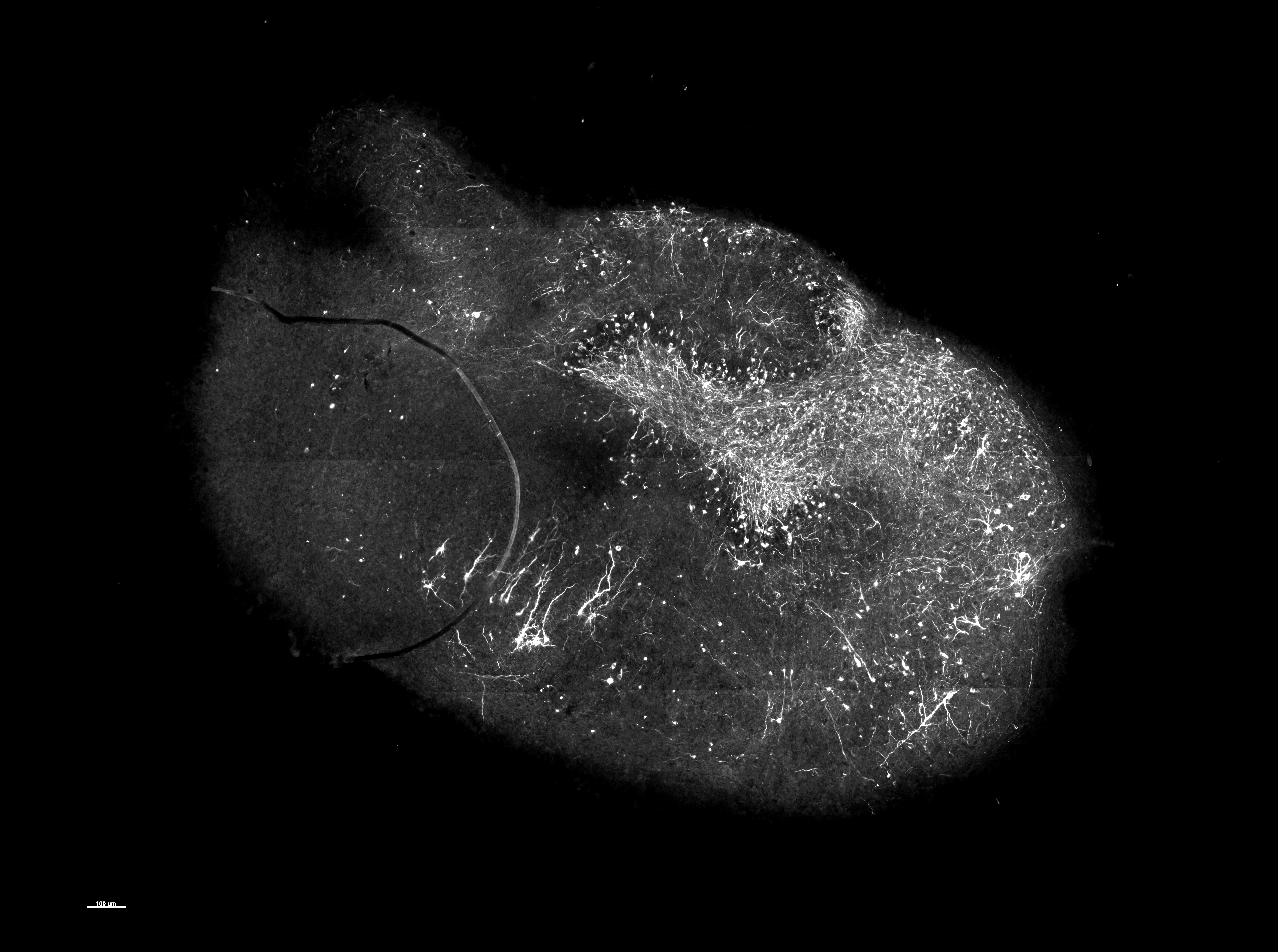

Supplement: Supplementary file 8 — Source Data for Figure 5 [file EMMM-13-e14745-s008.zip › Fig5/5C/20180425_LAG3-BLIND-1-r2s2_ThioS-FITC-700ms_pS129-Cy3-500ms_DAPI-30ms_10x_(DAPI+FITC+Cy5 5).TIF (red).tif]

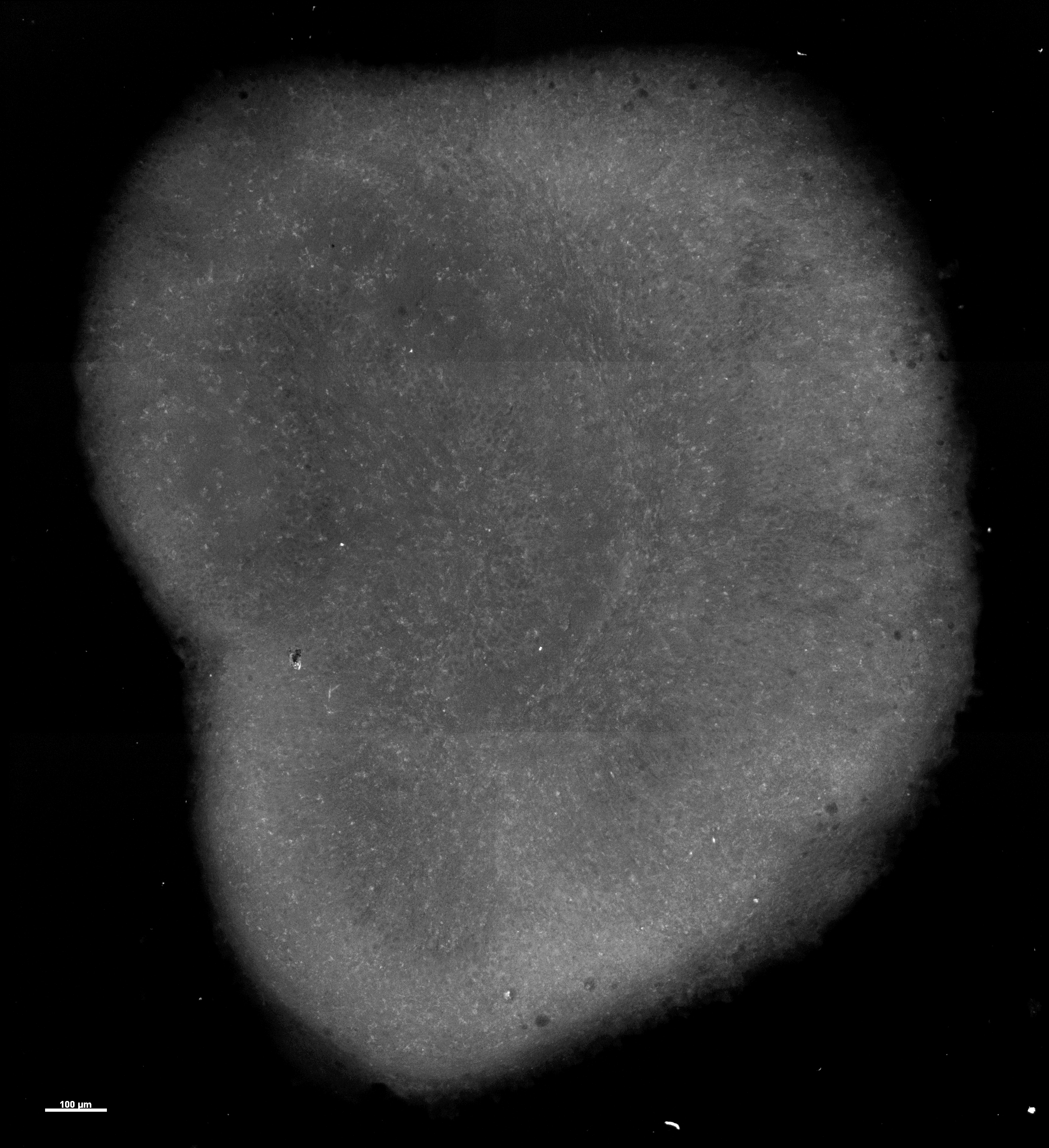

Supplement: Supplementary file 8 — Source Data for Figure 5 [file EMMM-13-e14745-s008.zip › Fig5/5C/20171207_lag3-exp_6-R2S2_ThioS+pS129-488_10x_(FITC).TIF (green).tif]

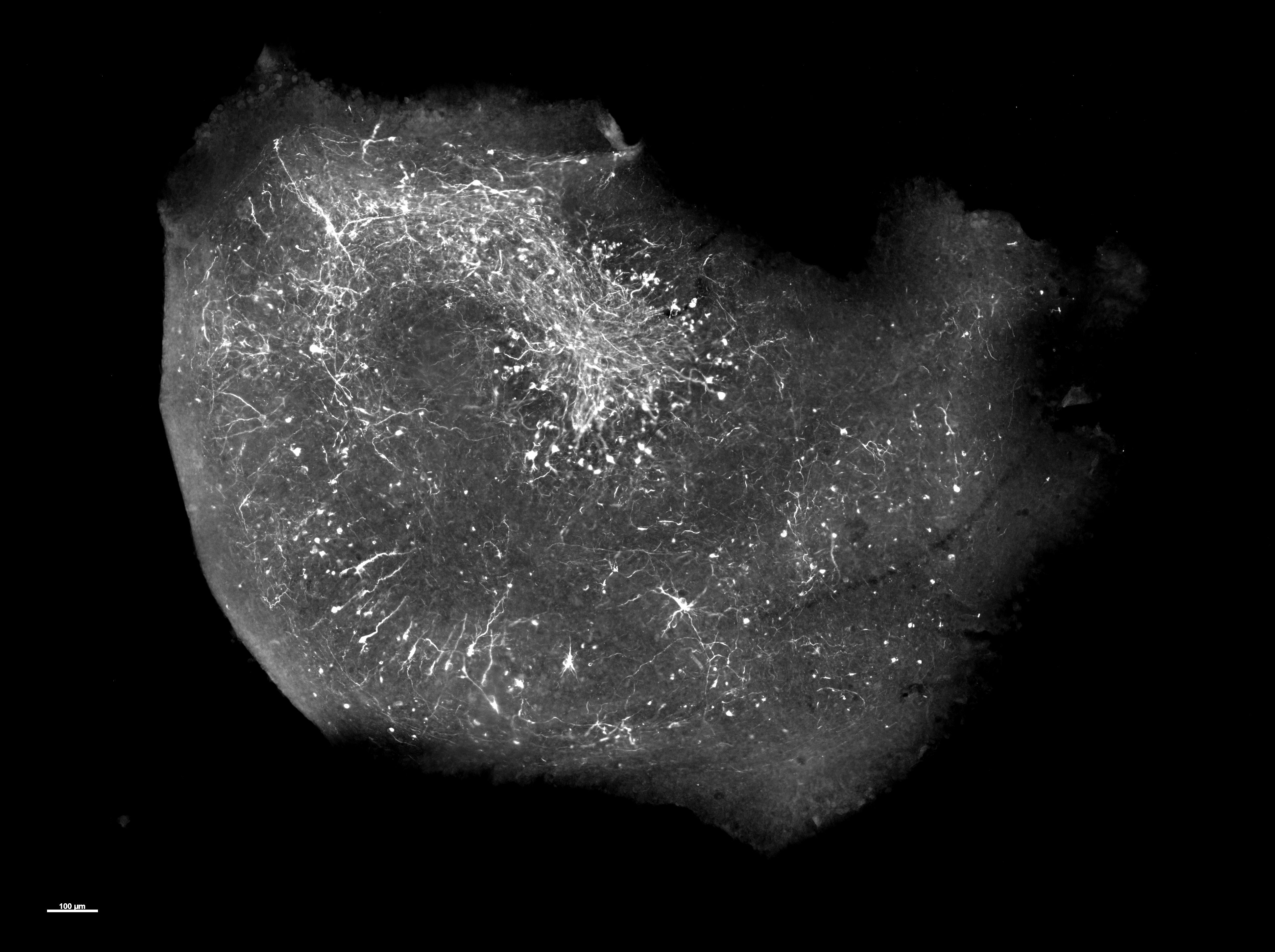

Supplement: Supplementary file 8 — Source Data for Figure 5 [file EMMM-13-e14745-s008.zip › Fig5/5C/20180425_LAG3-BLIND-4-r1s3_ThioS-FITC-700ms_pS129-Cy3-500ms_DAPI-30ms_10x_(DAPI+FITC+Cy5 5).TIF (red).tif]

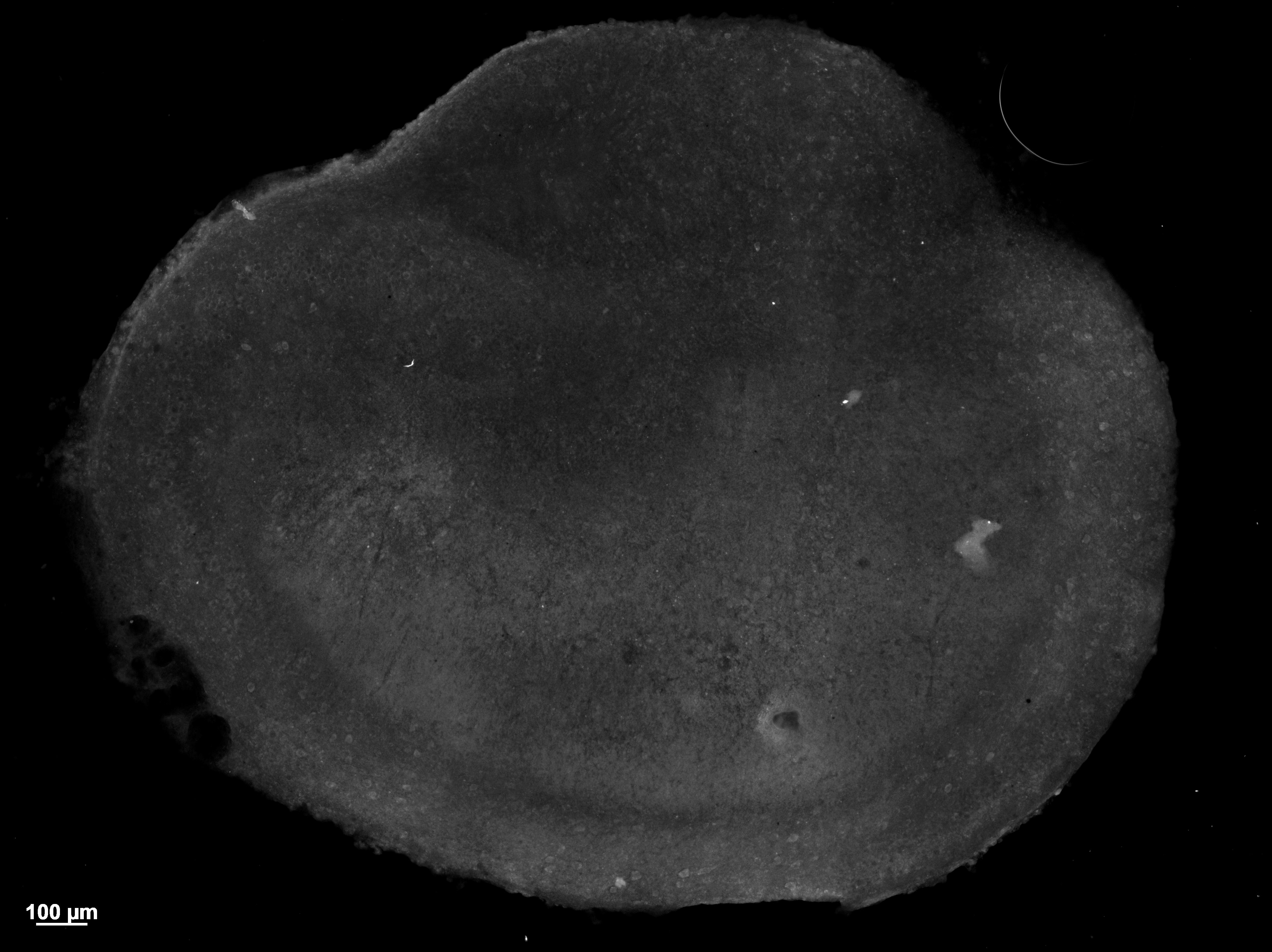

Supplement: Supplementary file 8 — Source Data for Figure 5 [file EMMM-13-e14745-s008.zip › Fig5/5D/20170721_hsc21-5_untr_ThioS_3_10xtg-untr.jpg]

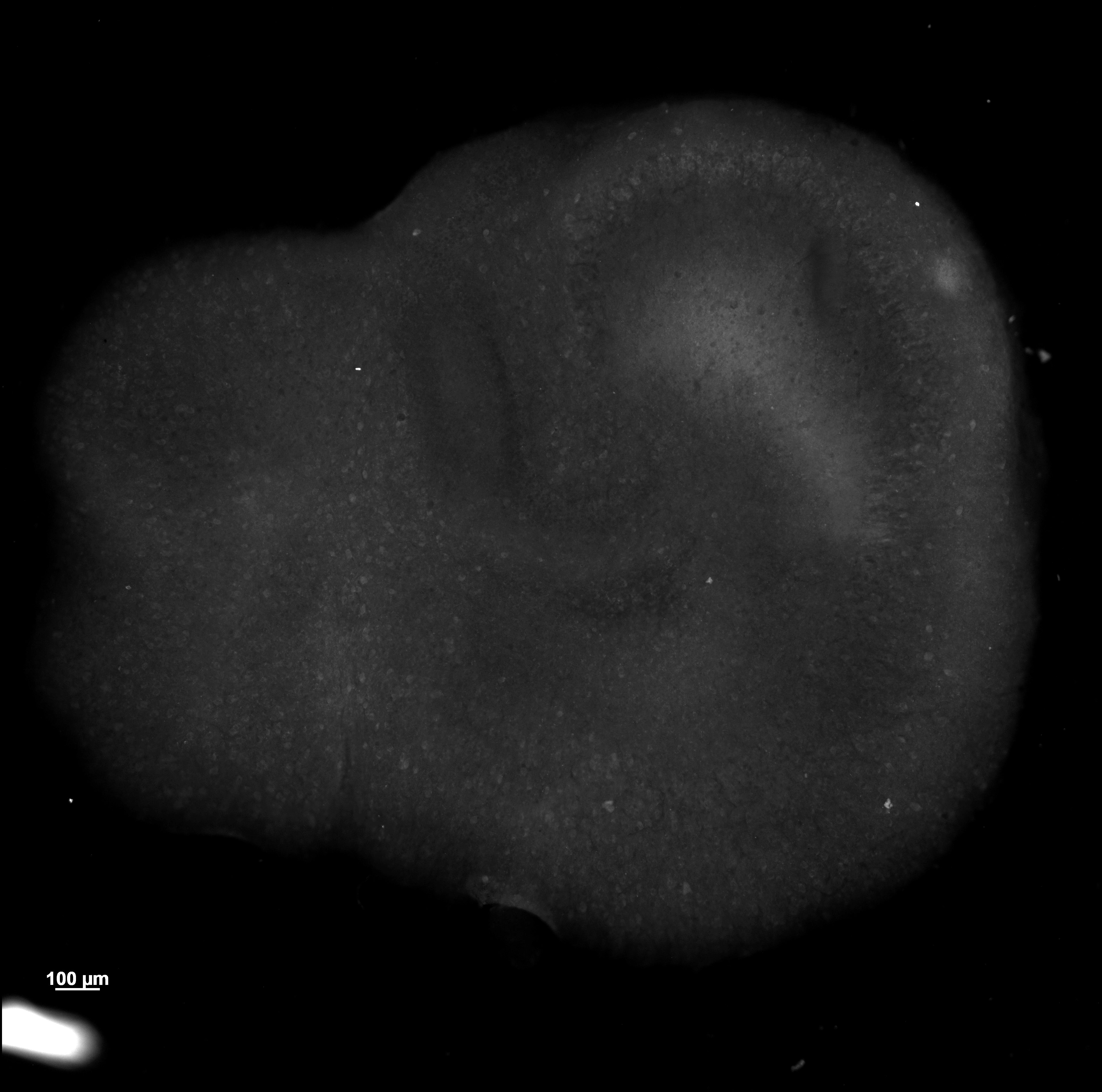

Supplement: Supplementary file 8 — Source Data for Figure 5 [file EMMM-13-e14745-s008.zip › Fig5/5D/20170721_hsc21-4_untr_ThioS_2_10x_wt-untr.jpg]

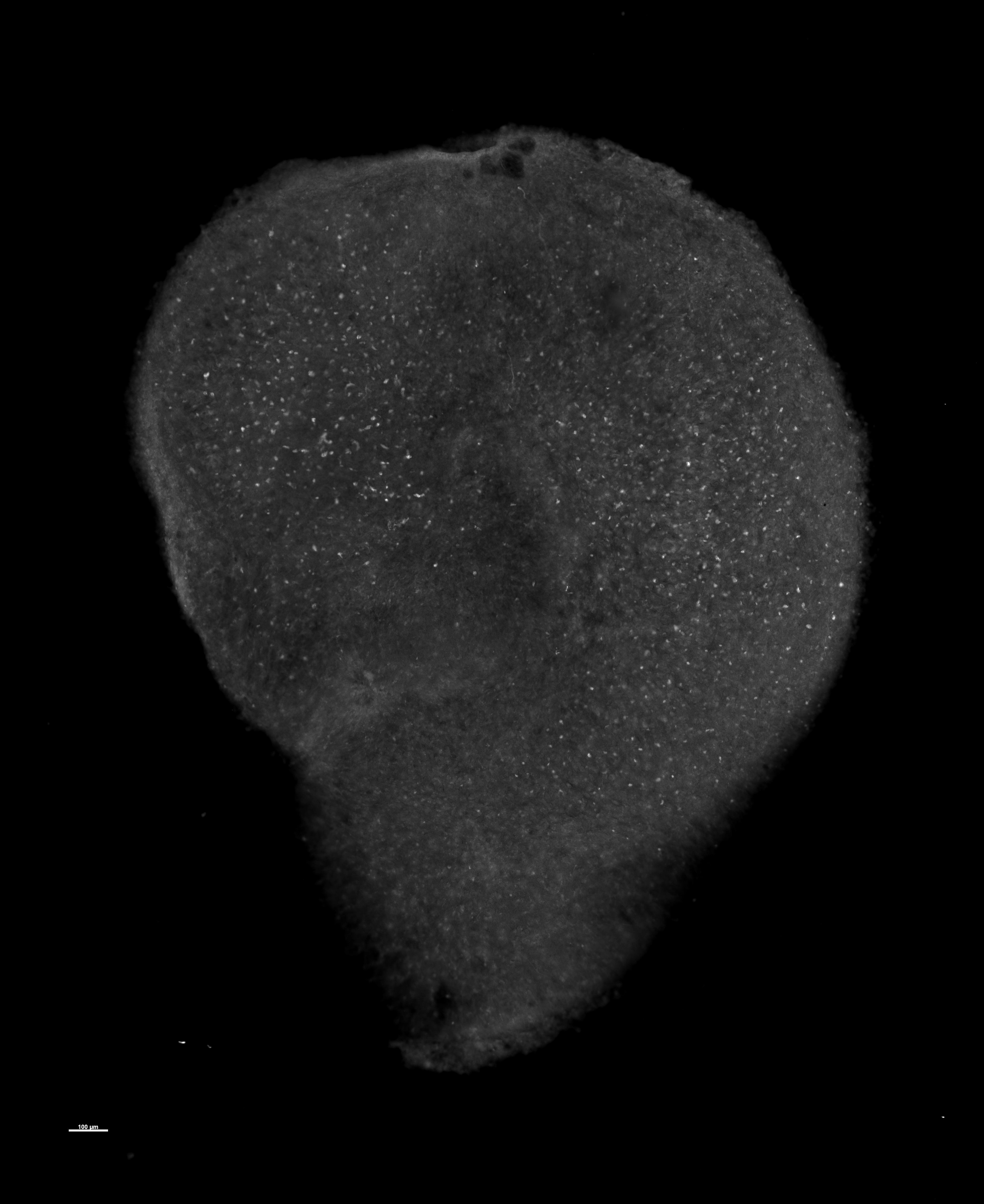

Supplement: Supplementary file 8 — Source Data for Figure 5 [file EMMM-13-e14745-s008.zip › Fig5/5D/20180425_LAG3-BLIND-4-r6s3_ThioS-FITC-700ms_pS129-Cy3-500ms_DAPI-30ms_10x_(DAPI+FITC+Cy5 5).TIF (green)_med.jpg]

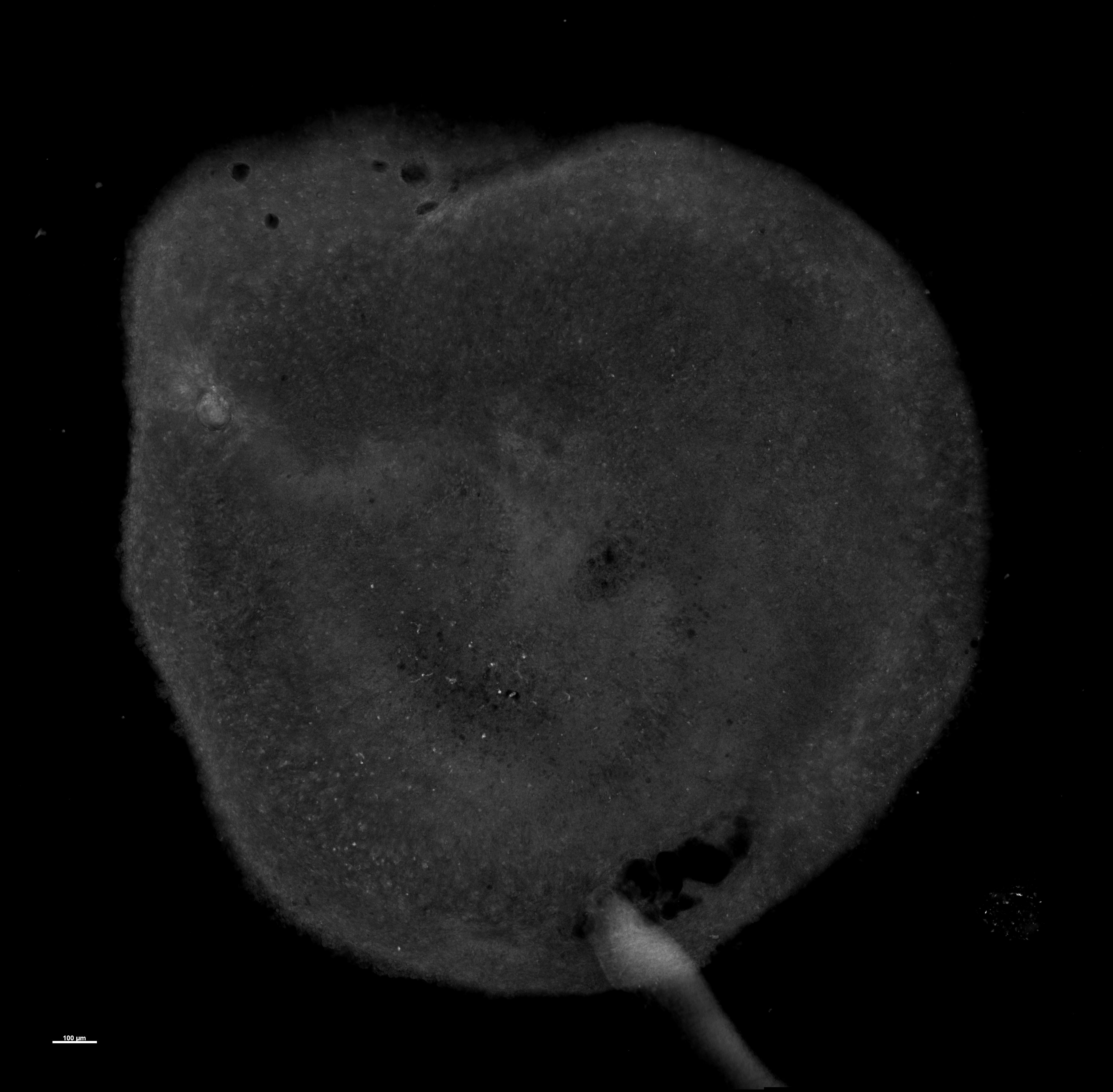

Supplement: Supplementary file 8 — Source Data for Figure 5 [file EMMM-13-e14745-s008.zip › Fig5/5D/20180426_LAG3-BLIND-9-r2s3_ThioS-FITC-700ms_pS129-Cy3-500ms_DAPI-30ms_10x_(DAPI+FITC+Cy5 5).TIF (green)_med.jpg]

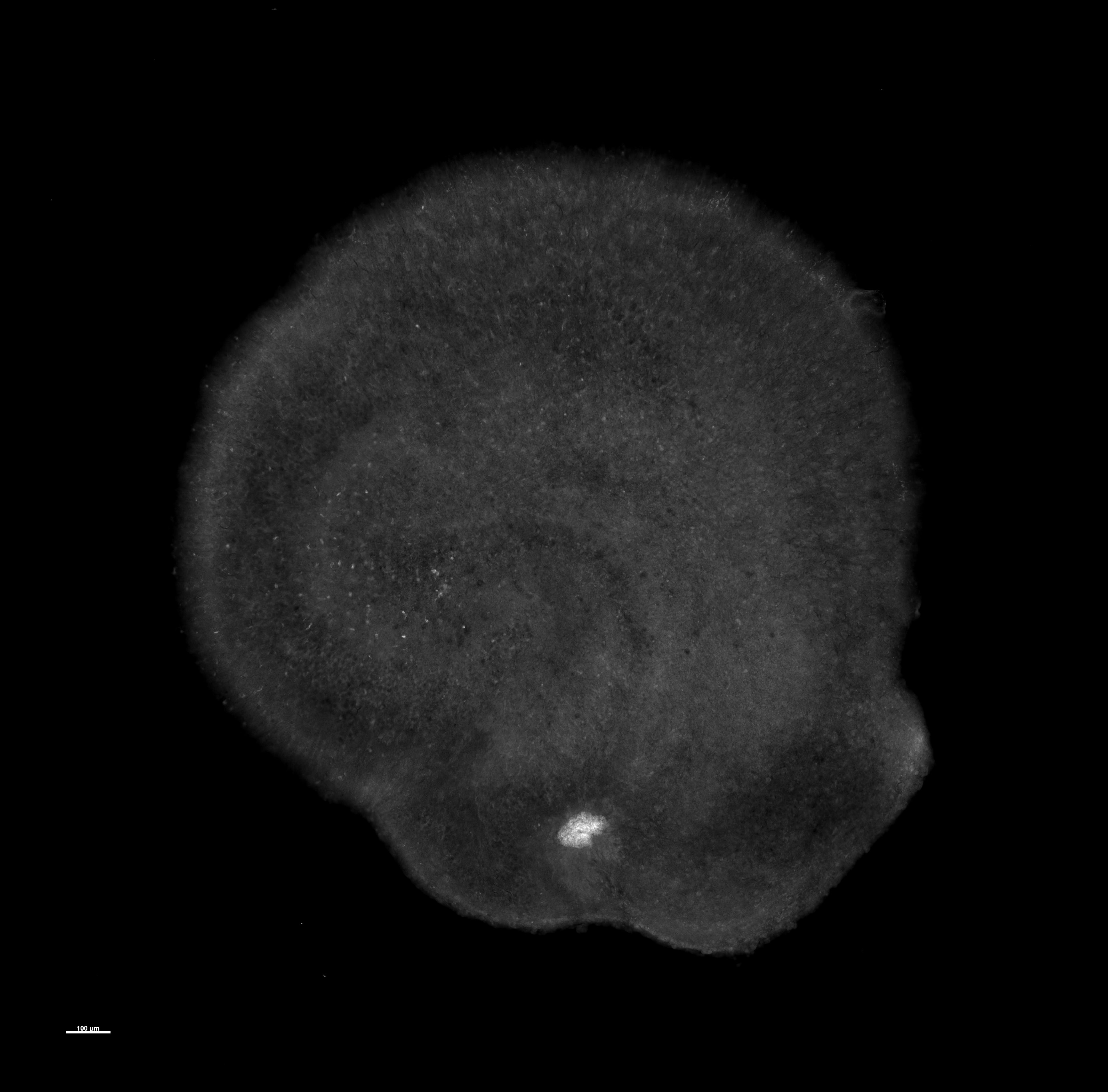

Supplement: Supplementary file 8 — Source Data for Figure 5 [file EMMM-13-e14745-s008.zip › Fig5/5D/20180426_LAG3-BLIND-7-r6s3_ThioS-FITC-700ms_pS129-Cy3-500ms_DAPI-30ms_10x_(DAPI+FITC+Cy5 5).TIF (green)_med.jpg]

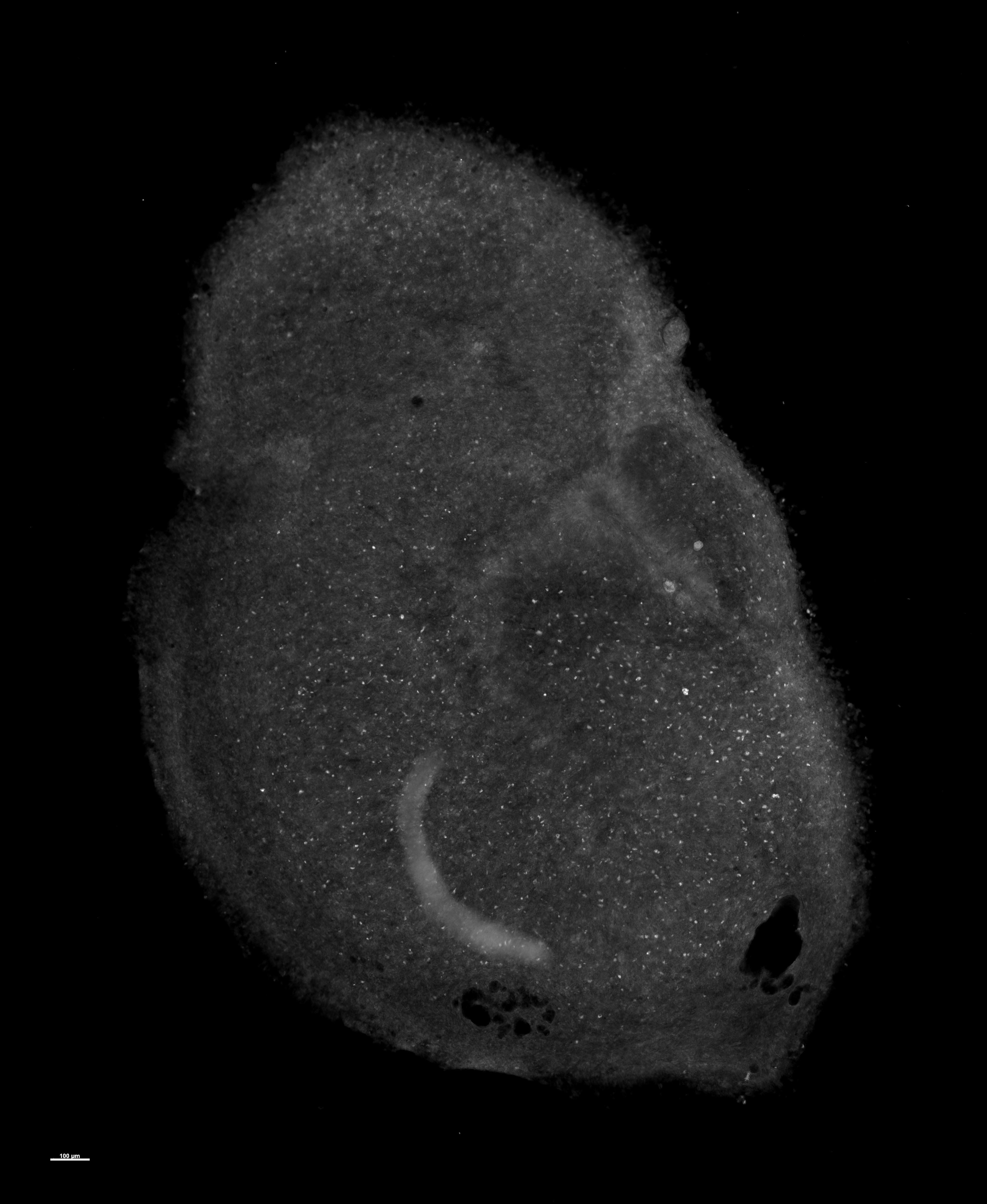

Supplement: Supplementary file 8 — Source Data for Figure 5 [file EMMM-13-e14745-s008.zip › Fig5/5D/20180426_LAG3-BLIND-8-r1s2_ThioS-FITC-700ms_pS129-Cy3-500ms_DAPI-30ms_10x_(DAPI+FITC+Cy5 5).TIF (green)_med.jpg]

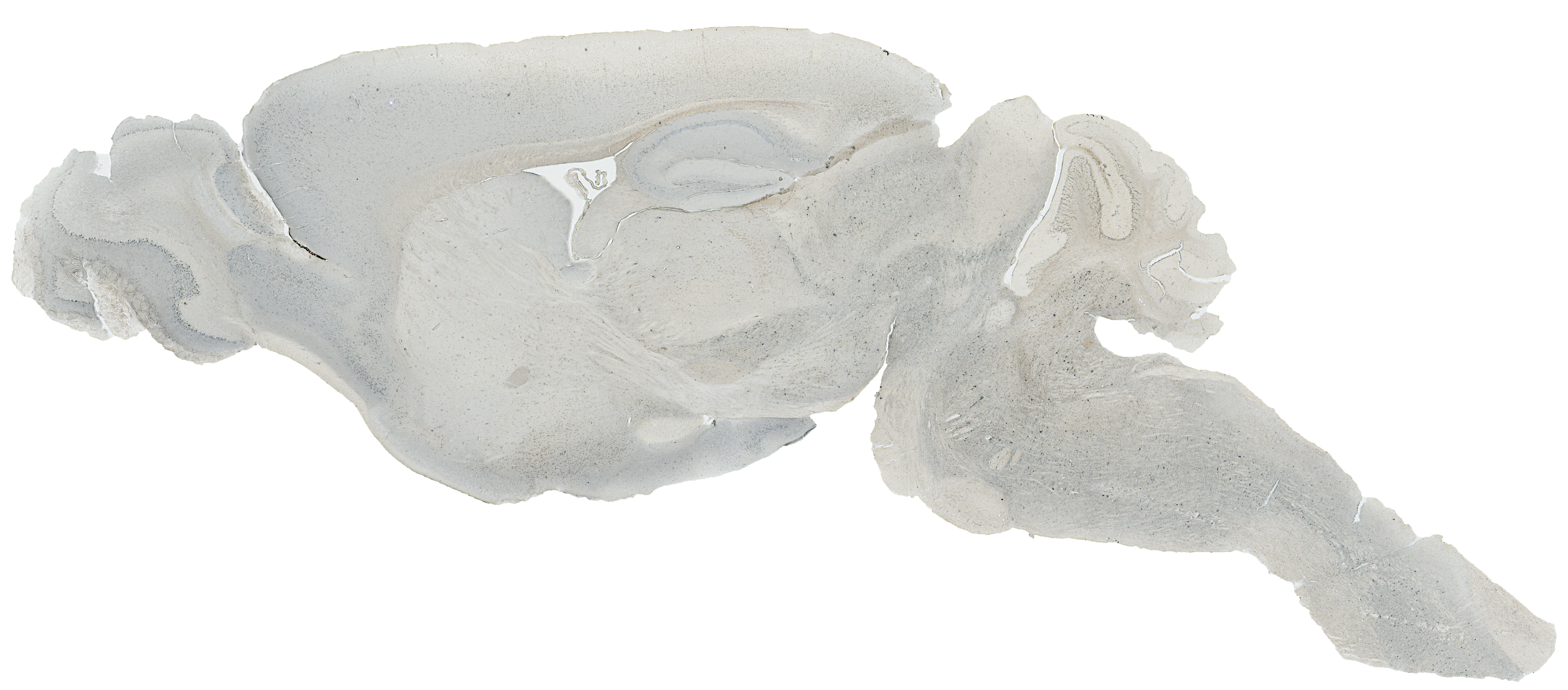

Supplement: Supplementary file 8 — Source Data for Figure 5 [file EMMM-13-e14745-s008.zip › Fig5/5B/right panel (367#49)/IP-ConvertImage-06.png]

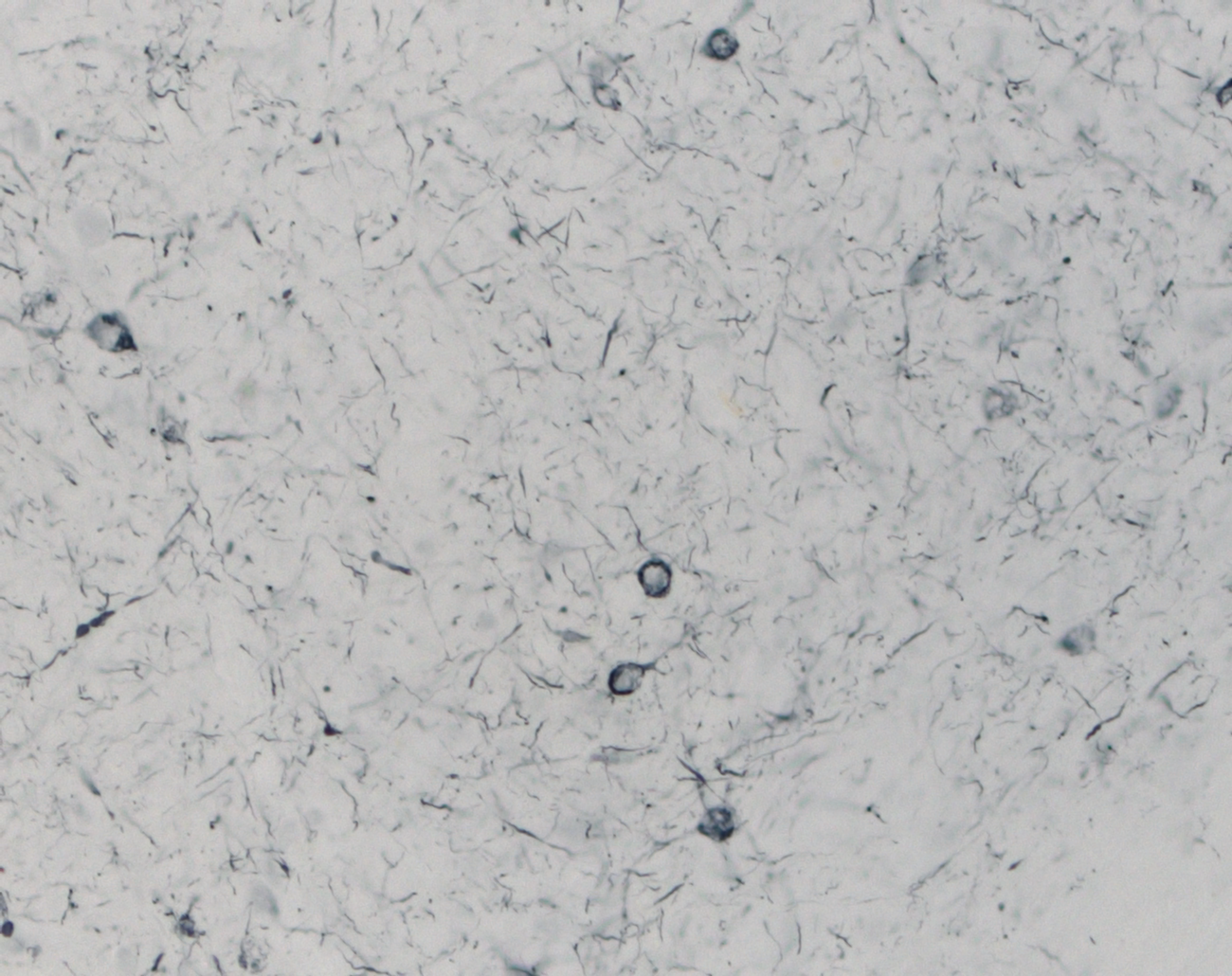

Supplement: Supplementary file 8 — Source Data for Figure 5 [file EMMM-13-e14745-s008.zip › Fig5/5B/right panel (367#49)/367_49-pS129-midbrain-20x-2.tif]

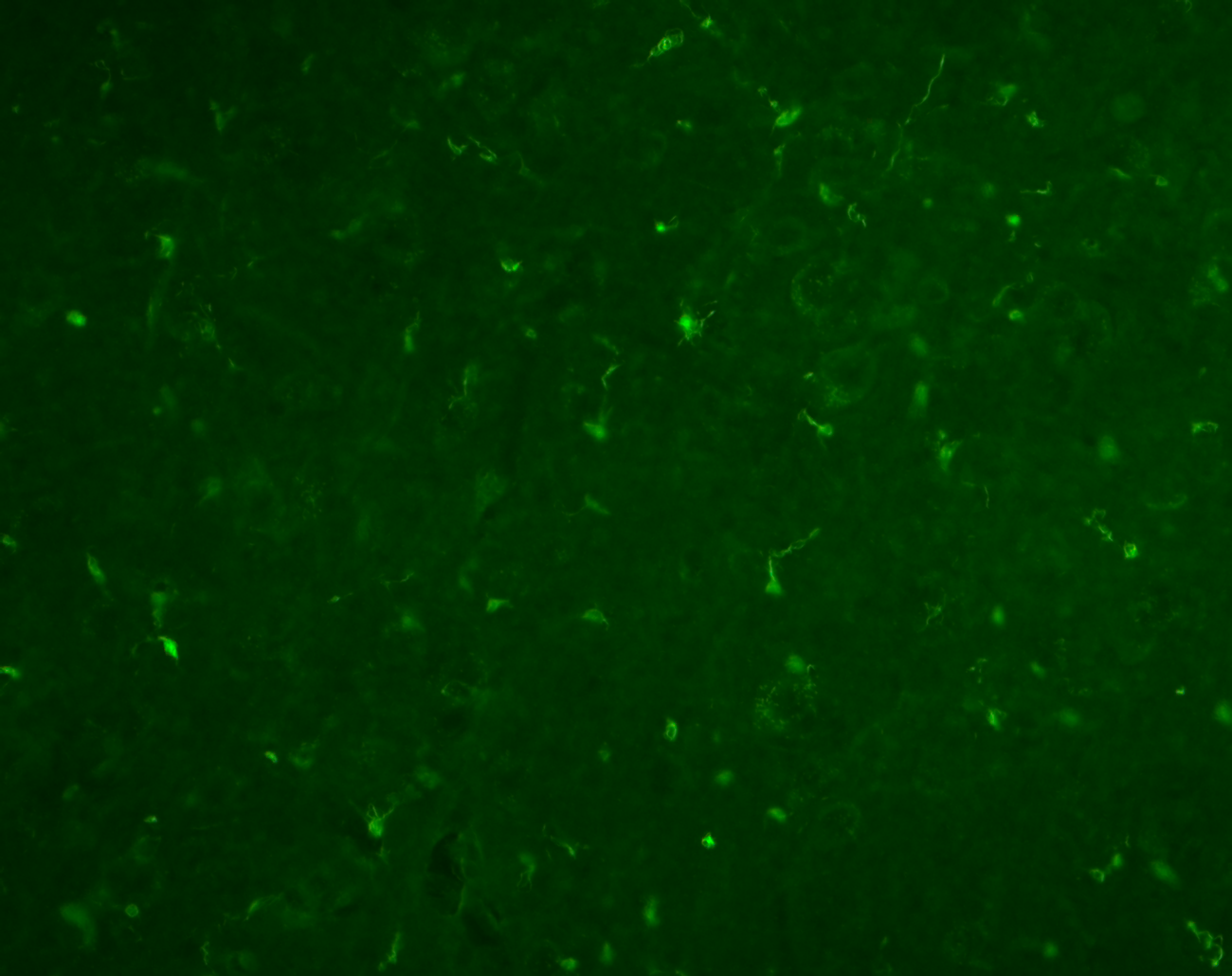

Supplement: Supplementary file 8 — Source Data for Figure 5 [file EMMM-13-e14745-s008.zip › Fig5/5B/right panel (367#49)/367-49 midbrain 20x_c0.tif]

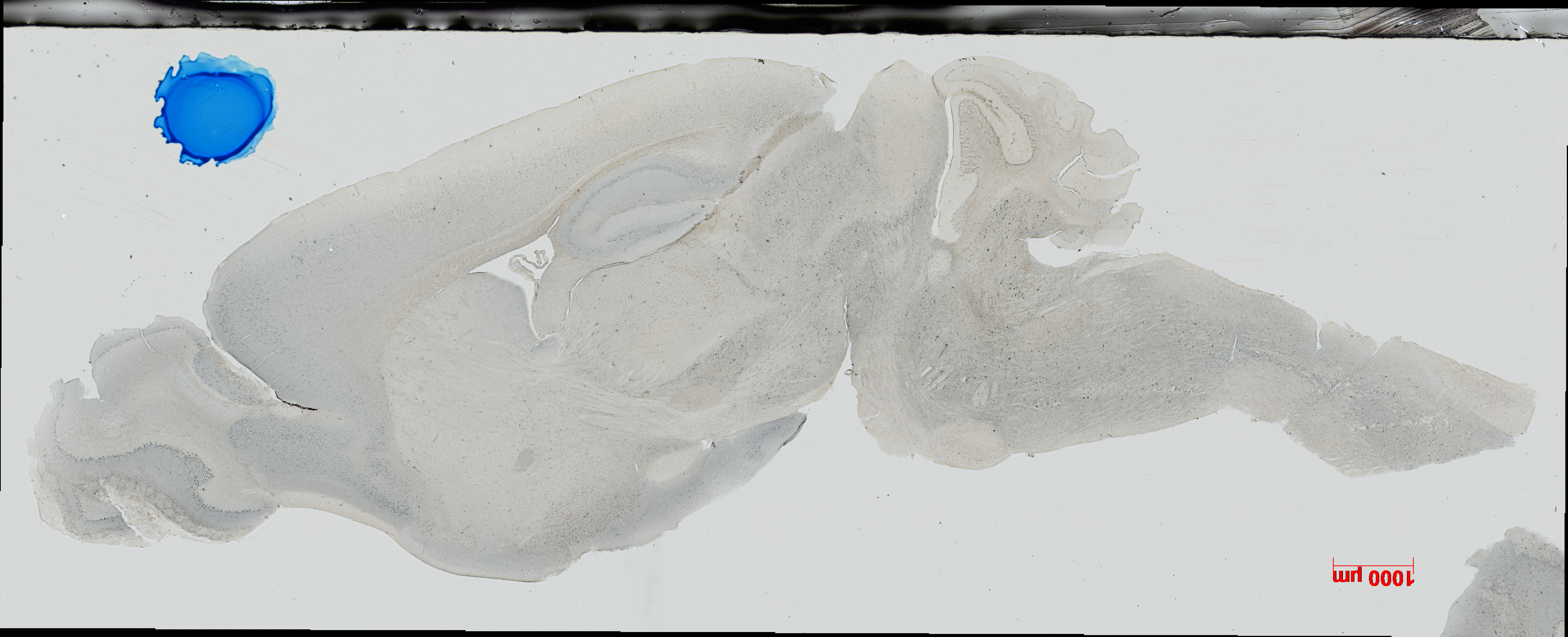

Supplement: Supplementary file 8 — Source Data for Figure 5 [file EMMM-13-e14745-s008.zip › Fig5/5B/right panel (367#49)/IP-ConvertImage-06_scale.tif]

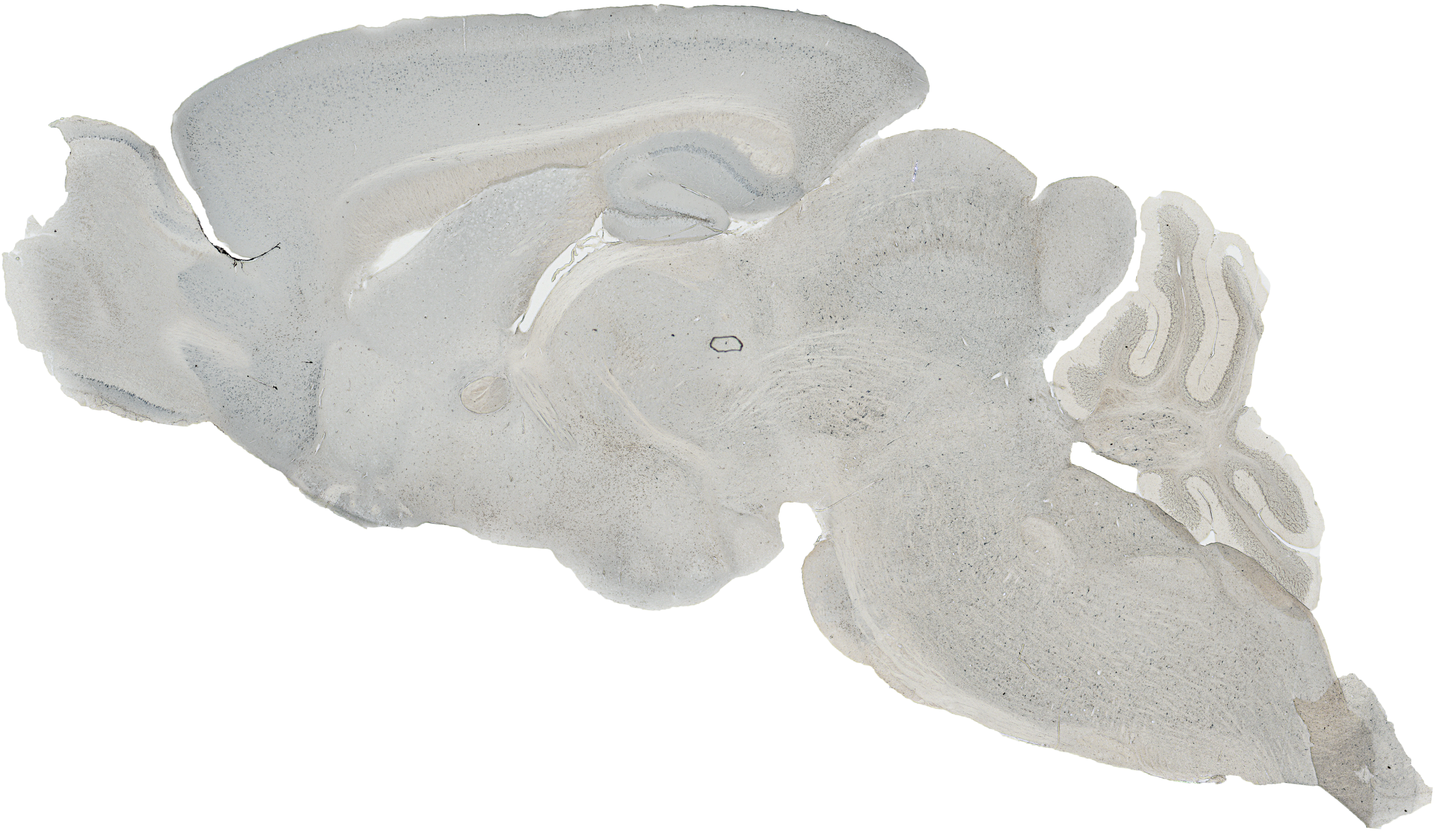

Supplement: Supplementary file 8 — Source Data for Figure 5 [file EMMM-13-e14745-s008.zip › Fig5/5B/left (367#103)/IP-ConvertImage-13.png]

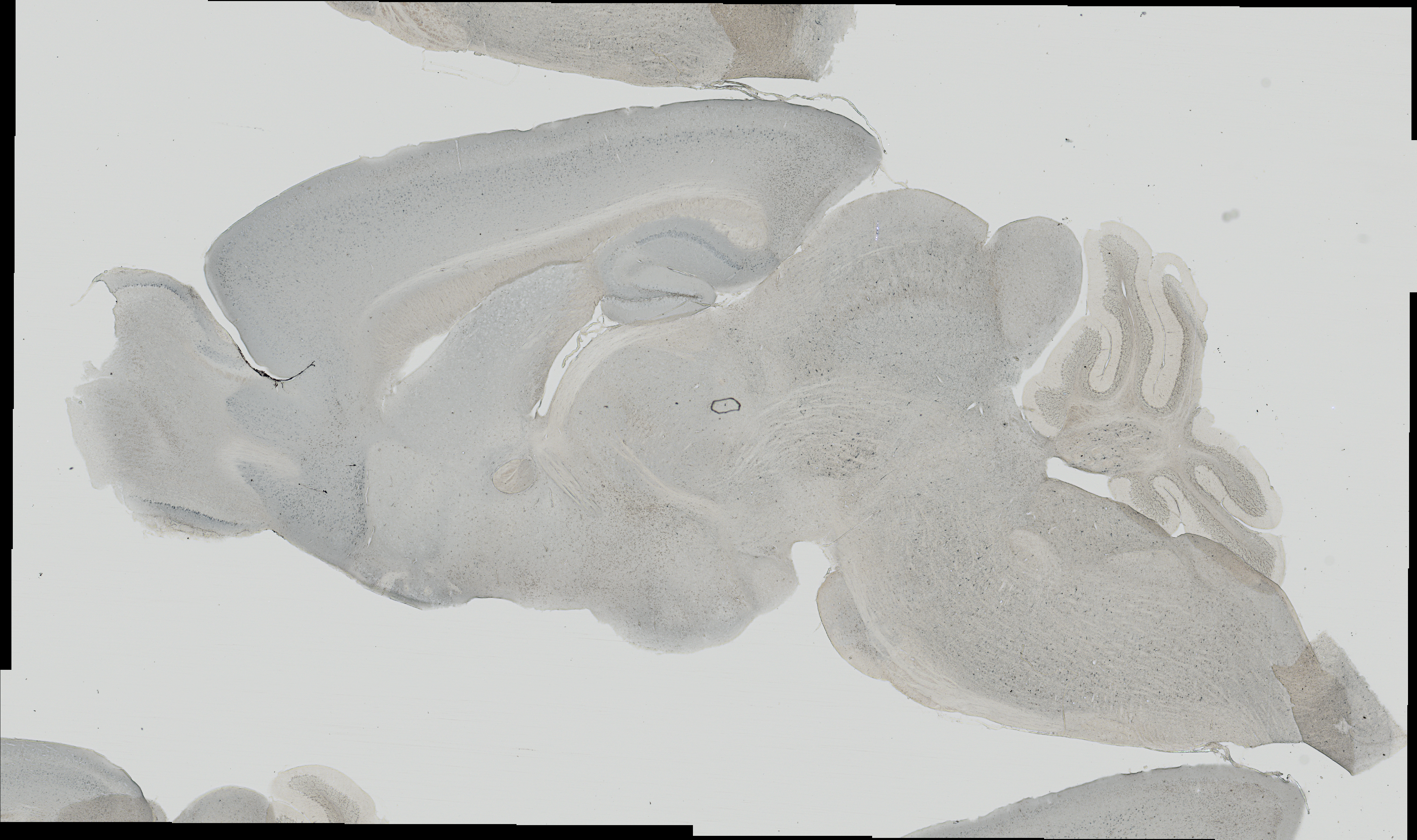

Supplement: Supplementary file 8 — Source Data for Figure 5 [file EMMM-13-e14745-s008.zip › Fig5/5B/left (367#103)/IP-ConvertImage-13.tif]

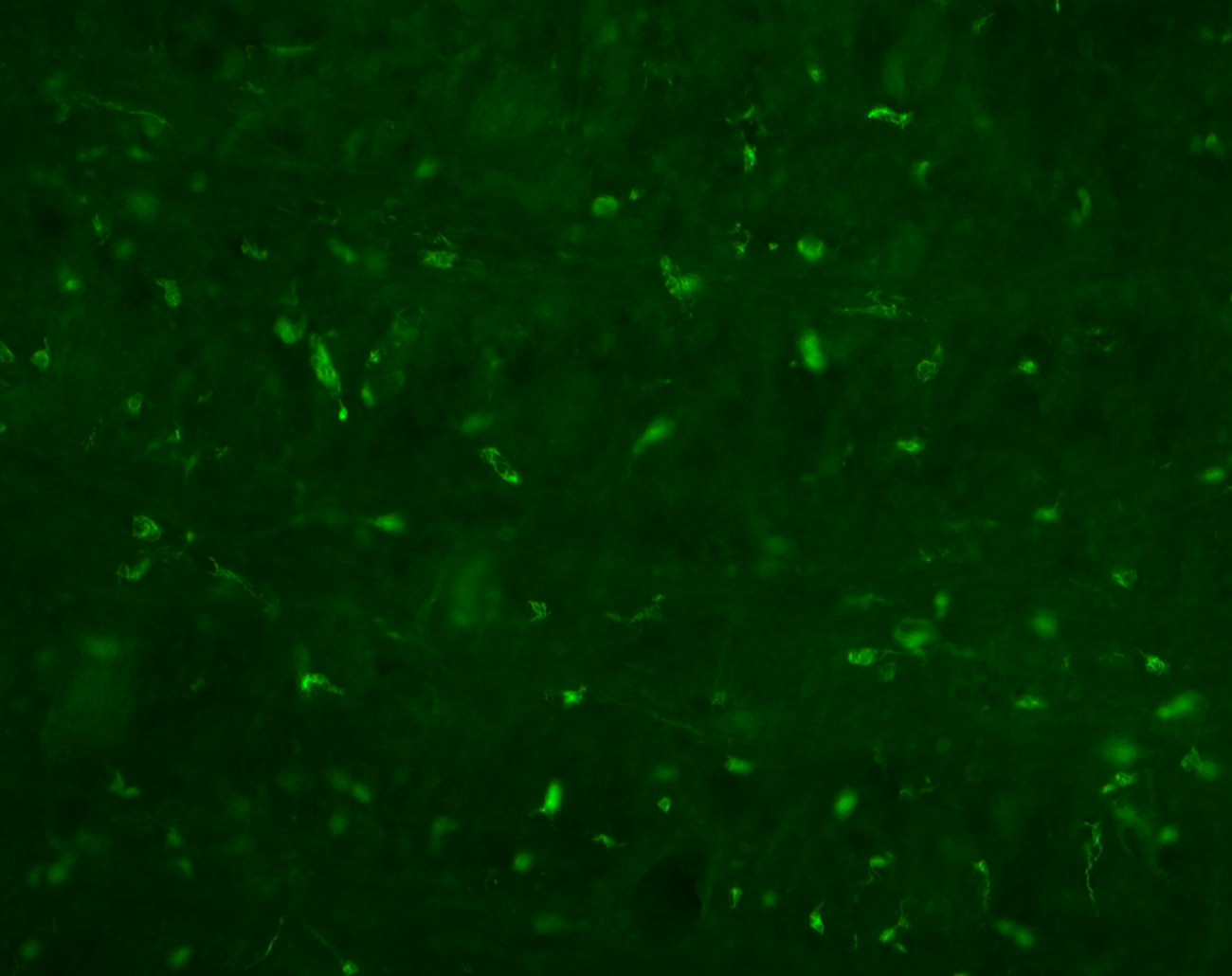

Supplement: Supplementary file 8 — Source Data for Figure 5 [file EMMM-13-e14745-s008.zip › Fig5/5B/left (367#103)/367-103 midbrain 20x_c0.tif]

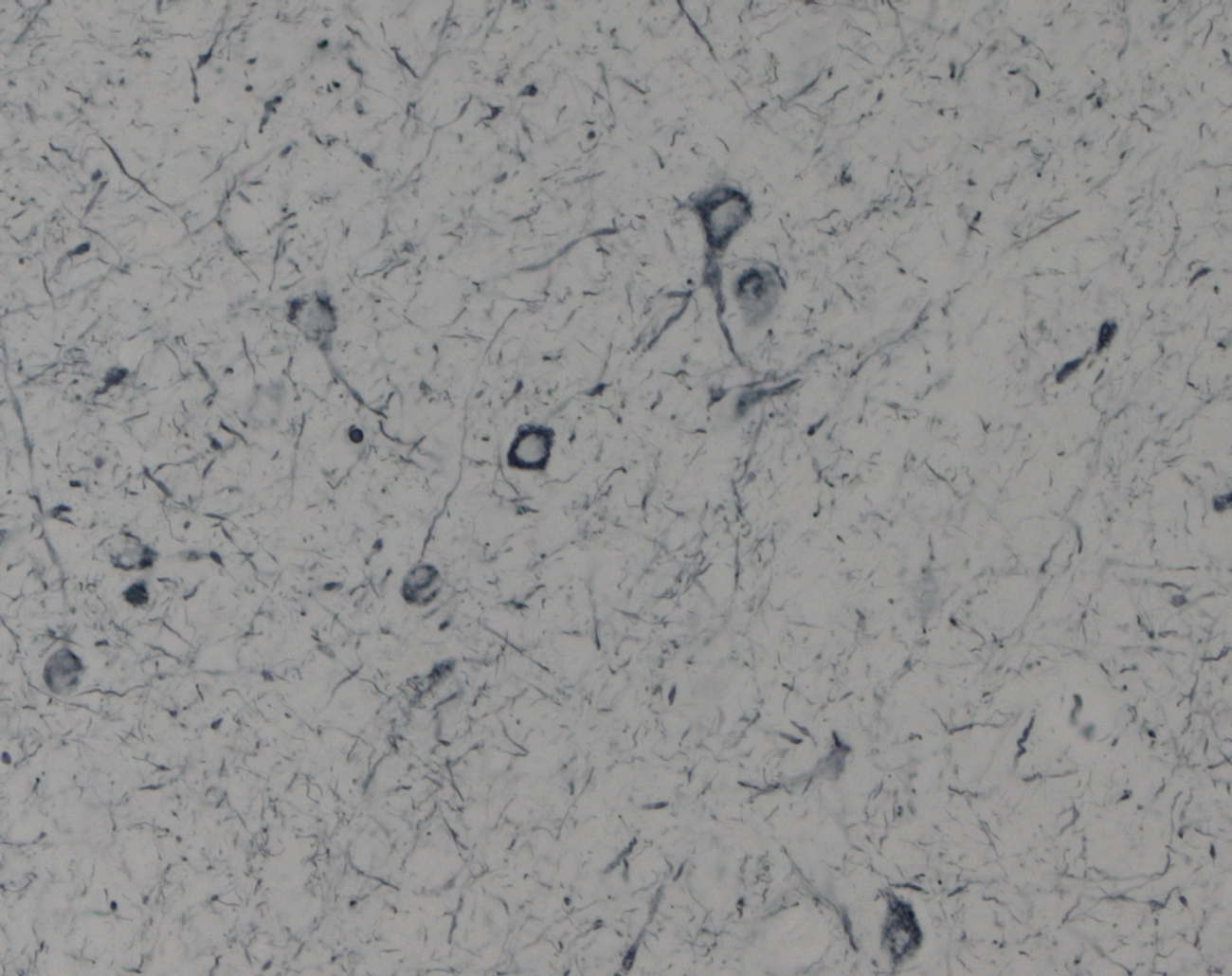

Supplement: Supplementary file 8 — Source Data for Figure 5 [file EMMM-13-e14745-s008.zip › Fig5/5B/left (367#103)/367_103-s129p-20x-midbrain.tif]
